# Supplementary material for: LolA and LolB are conserved in Bacteroidota and are crucial for gliding motility and Type IX secretion
Source: Commun Biol. 2025 Mar 6;8:376. doi: 10.1038/s42003-025-07817-2 (PMC11885536; doi:10.1038/s42003-025-07817-2)
Supplement: Supplementary file 2 — Supplementary Information [file 42003_2025_7817_MOESM2_ESM.pdf]

Supplementary Information

LolA and LolB are conserved in Bacteroidota and are crucial for gliding motility and Type IX secretion.

Tom De Smet, Elisabeth Baland, Fabio Giovannercole, Julien Mignon, Laura Lizen, Rémy Dugauquier, Frédéric Lauber, Marc Dieu, Gipsi Lima-Mendez, Catherine Michaux, Damien Devos and Francesco Renzi

Supplementary Figures

- **Supplementary Figure 1.** Comparison of per-residue hydrophobicity between *E. coli* and *F. johnsoniae* LolA and LolB homologs.
- **Supplementary Figure 2.** Comparison of charge state and distribution between *E. coli* and *F. johnsoniae* LolA and LolB homologs.
- **Supplementary Figure 3.** Transmission Electron Microscopy micrographs of WT and mutant *F. johnsoniae* cells grown for 16 hours in CYE liquid medium.
- **Supplementary Figure 4.** Phase-contrast microscopy images of WT and mutant *F. johnsoniae* cells grown for 16 hours in CYE and MM liquid media.
- **Supplementary Figure 5.** Deletion of *lolA* or *lolB* in *E. coli MG1655* complemented with *lolA* or *lolB*.
- **Supplementary Figure 6.** Uncropped blot image of Figure 6d

Supplementary Tables

- **Supplementary Table 1.** Composition in hydrophobic, aromatic, negatively and positively charged residues of the Lol protein homologs from *E. coli* (LolA and LolB) and *F. johnsoniae* (LolA1, LolA2, LolA3, LolB1, and LolB2).
- **Supplementary Table 2.** Detection of LolB1 variants in *F. johnsoniae* by mass spectrometry.
- **Supplementary Table 3.** Detection of *E. coli* LolA and LolB in *F. johnsoniae* and of *F. johnsoniae* LolA1 and LolB1 in *E. coli* by mass spectrometry.
- **Supplementary Table 4.** LolA and LolB homologs in several Bacteroidota species.
- **Supplementary Table 5.** Gliding and T9SS-related proteins detected in the OM of the *lolA1* and *lolB1* mutants.
- **Supplementary Table 6.** Bacterial strains used in this study.
- **Supplementary Table 7.** Plasmids used in this study.
- **Supplementary Table 8.** Oligonucleotides used in this study.

Supplementary References

Supplementary Figures

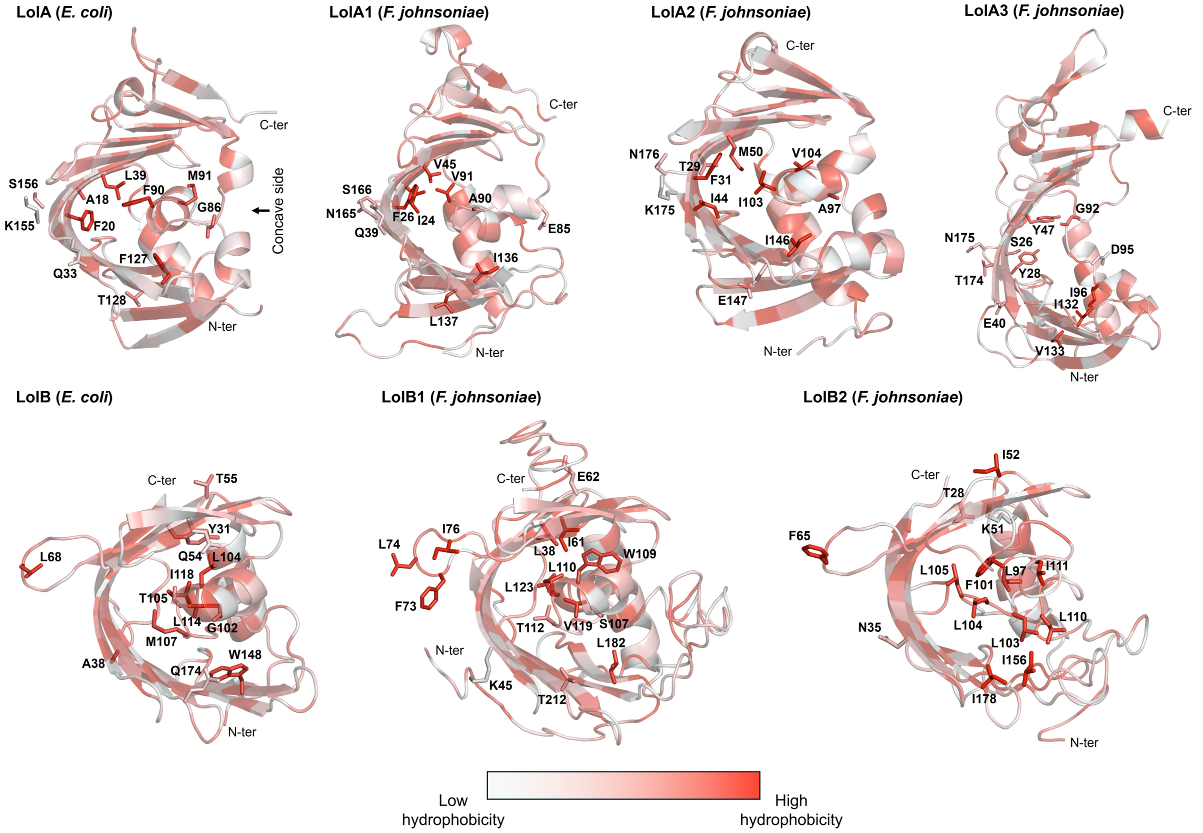

Supplementary Figure 1. **Comparison of per-residue hydrophobicity between *E. coli* and *F. johnsoniae* LolA and LolB homologs.** Hydrophobicity scores, ranging from hydrophilic (white) to highly hydrophobic (red), were computed using the Eisenberg's scale and projected on the crystallized LolA (PDB entry: 1UA8) and LolB (PDB entry: 1IWM) structures, as well as LolA1, LolA2, LolA3, LolB1, and LolB2 models represented as cartoon from the barrel opening view. On each structure, the N- and C-terminal positions are indicated. Identified amino acids relevant to lipoprotein binding in *E. coli* and their equivalents in *F. johnsoniae* are shown as stick with their associated labelled position. Residues in the upward loop of LolB proteins are also highlighted as stick with their associated labelled position.

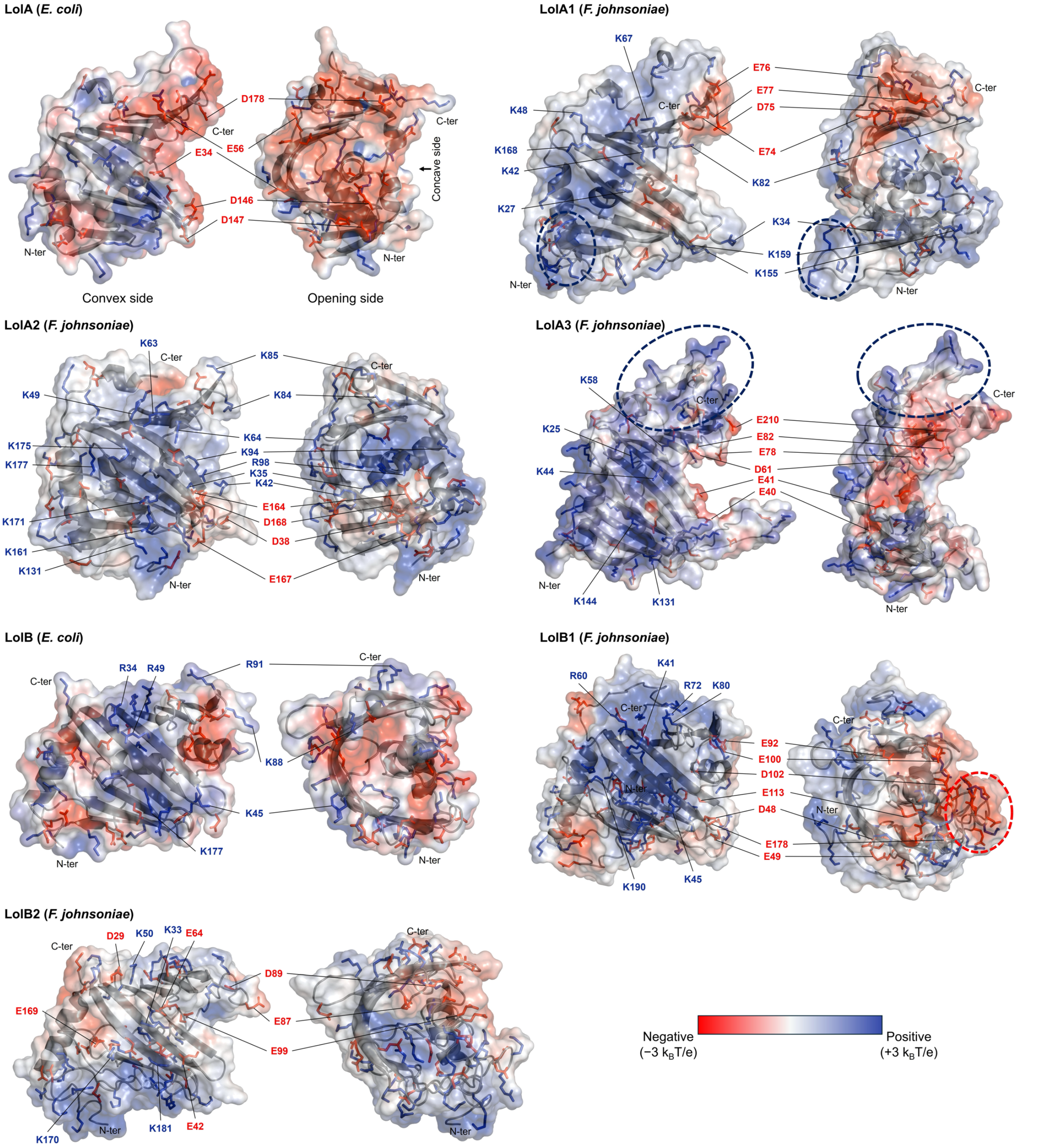

Supplementary Figure 2. **Comparison of charge state and distribution between *E. coli* and *F. johnsoniae* LolA and LolB homologs.** Poisson-Boltzmann electrostatic potentials, ranging from −3 k_BT/e (red) to +3 k_BT/e (blue), were mapped on the van der Waals surface of the crystallized LolA (PDB entry: 1UA8) and LolB (PDB entry: 1IWM) structures, as well as LolA1, LolA2, LolA3, LolB1, and LolB2 models represented as grey cartoon. On each structure, the N- and C-terminal positions are indicated. Negatively (aspartate and glutamate) and positively (lysine and arginine) charged residues are shown as red and blue stick, respectively. Relevant charged amino acids with respect to their position on the protein convex side or barrel opening have their position labelled. In LolA1, the positively charged loop extension between α-helix 3 and β-strand 7 is highlighted with a dashed dark blue ellipse. On the LolA3 panel, the positively charged C-terminal region is highlighted with a dashed dark blue ellipse. In LolB1 panel, the negatively charged loop extension between α-helix 3 and β-strand 7 is highlighted with a dashed red ellipse.

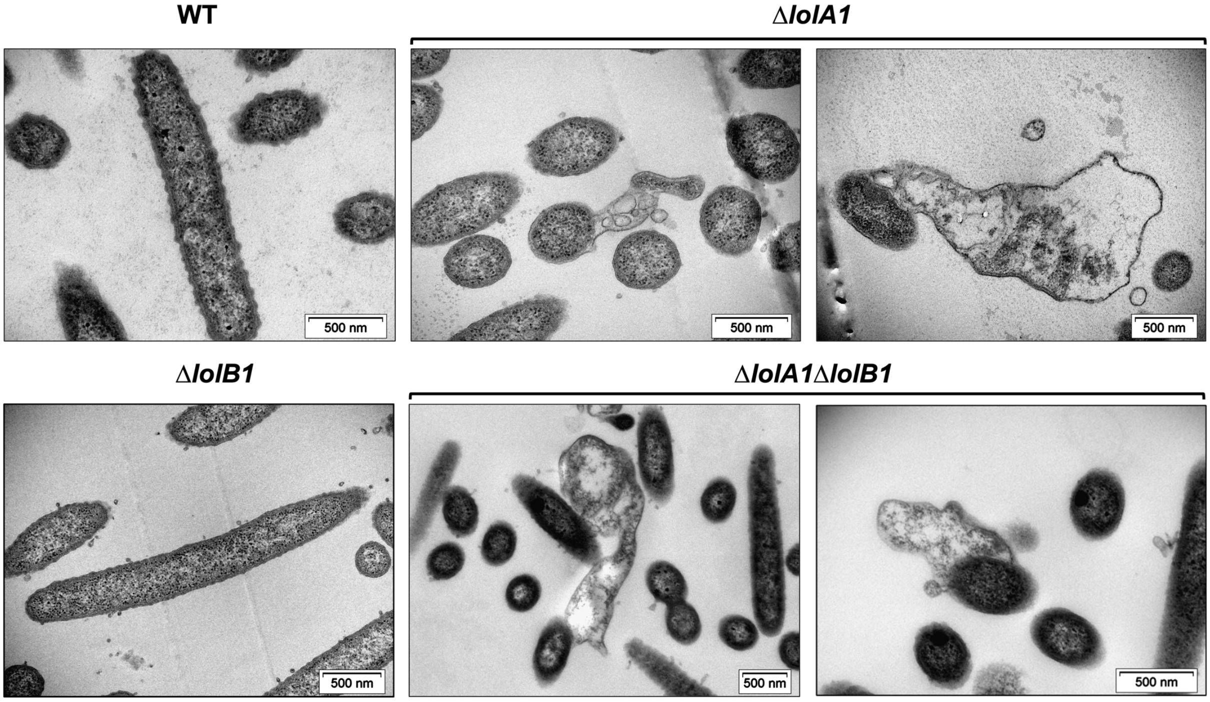

Supplementary Figure 3. Transmission Electron Microscopy micrographs of WT and mutant *F. johnsoniae* cells grown for 16 hours in CYE liquid medium.

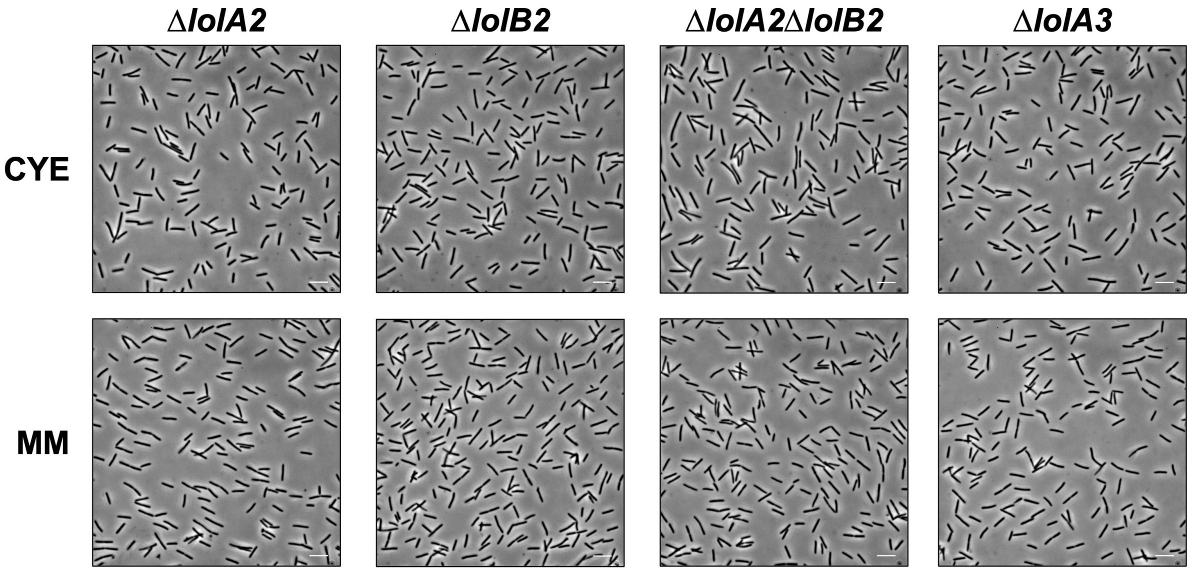

Supplementary Figure 4. Phase-contrast microscopy images of WT and mutant *F. johnsoniae* cells grown for 16 hours in CYE and MM liquid media (bar = 5 µm).

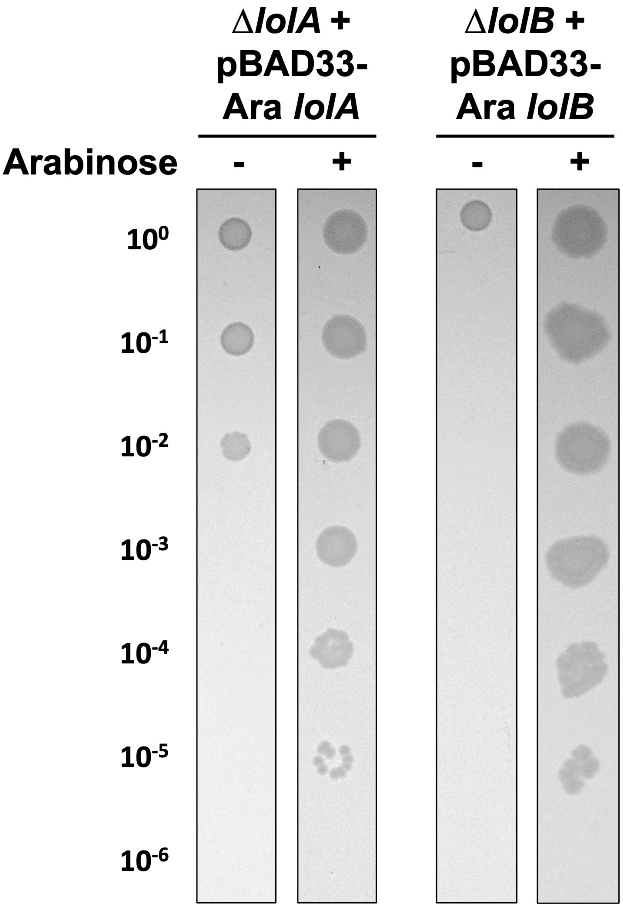

Supplementary Figure 5. Deletion of *lolA* or *lolB* in *E. coli MG1655* complemented with *lolA* or *lolB.* Serial dilution spots on LB agar plates supplemented with (+) or without (-) 0.2% arabinose of the *lolA* and *lolB* mutant strains expressing *E. coli lolA* or *lolB* in trans.

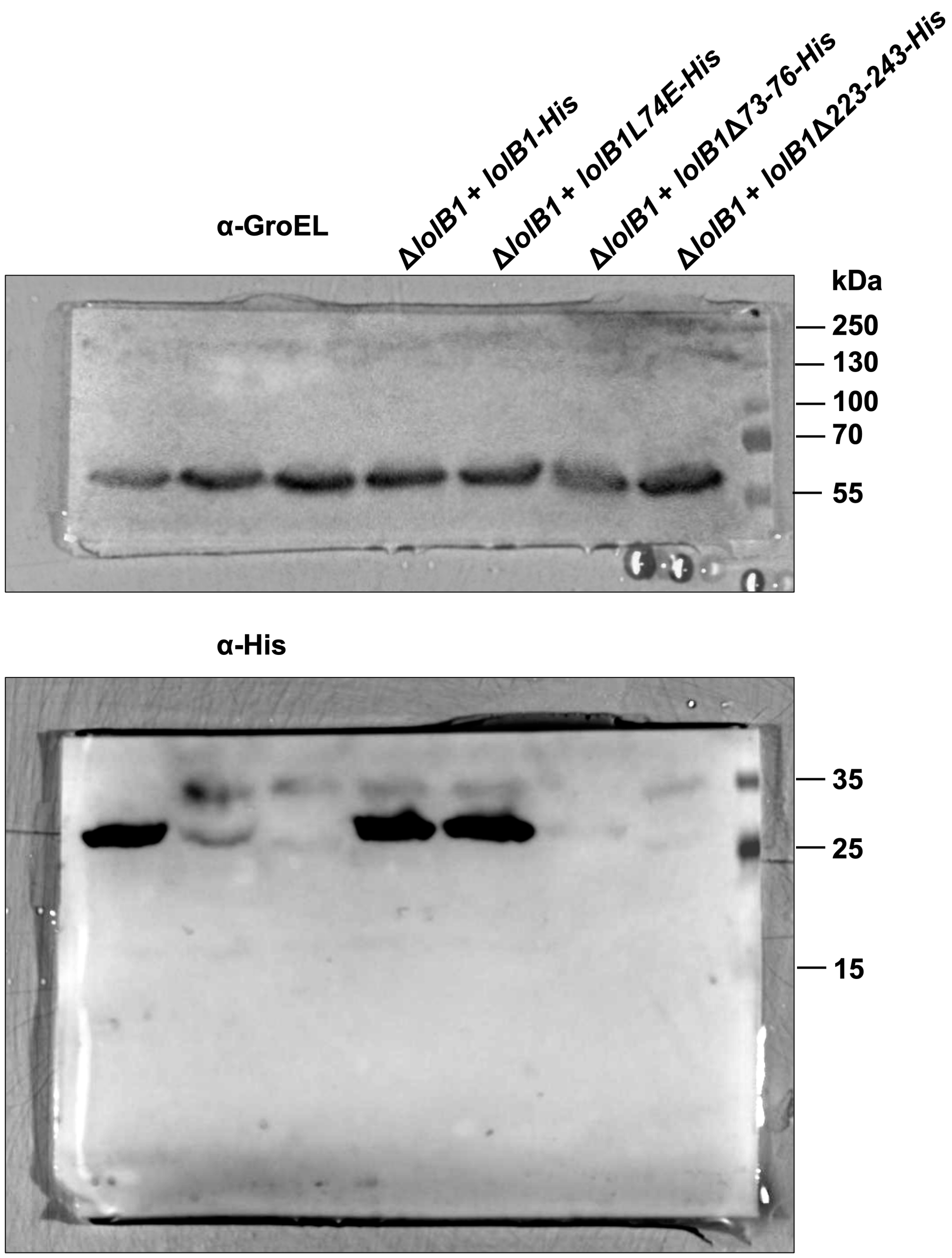

Supplementary Figure 6. Uncropped blot image of Figure 6d.

Supplementary Tables

Supplementary Table 1. Composition in hydrophobic, aromatic, negatively and positively charged residues of the Lol protein homologs from *E. coli* (LolA and LolB) and *F. johnsoniae* (LolA1, LolA2, LolA3, LolB1, and LolB2).

	Residue percentage (%)									
	**Hydrophobic**	**Aromatic**	**Ile/Leu**	**Val**	**Asp**	**Glu**	**Lys**	**Arg**	**−**	**+**
**LolA**	34.6	10.4	8.8	6.6	9.3	2.2	6.6	3.3	11.5	9.9
**LolA1**	35.6	9.8	13.4	8.2	6.7	4.1	14.4	1.0	10.8	15.4
**LolA2**	40.5	10.5	15.8	7.4	7.4	6.3	15.8	1.0	13.7	16.8
**LolA3**	39.7	14.2	14.6	7.3	4.6	5.9	13.2	1.8	10.5	15.0
**LolB**	34.4	9.7	12.9	4.3	7.5	3.2	6.4	5.4	10.7	11.8
**LolB1**	39.5	10.3	16.9	5.8	4.9	8.6	11.5	2.1	13.6	13.6
**LolB2**	37.4	12.1	18.4	4.2	6.3	8.4	14.2	2.1	14.7	16.3

Supplementary Table 2. Detection of LolB1 variants in *F. johnsoniae* by mass spectrometry.
Listed are the normalized total spectra number, percent coverage and protein identification probability of the proteins identified by mass spectrometry in the bacterial total lysates.

Strain	Description	Accession Number	Gene code	Normalized Total Spectra	Percentage coverage	Protein Identification Probability
F. johnsoniae UW101 Δ*lolB1* + pCP23-P*ermF-lolB1*$_{L74E}$	LolB1	A5FL25	*Fjoh_1066*	159	82%	100%
F. johnsoniae UW101 Δ*lolB1* + pCP23-P*ermF-lolB1*$_{\Delta 73\text{-}76}$	LolB1	A5FL25	*Fjoh_1066*	82	77%	100%
F. johnsoniae UW101 Δ*lolB1* + pCP23-P*ermF-lolB1*$_{\Delta 223\text{-}243}$	LolB1	A5FL25	*Fjoh_1066*	20	49%	100%

Supplementary Table 3. Detection of *E. coli* LolA and LolB in *F. johnsoniae* and of *F. johnsoniae* LolA1 and LolB1 in *E. coli* by mass spectrometry.
Listed are the normalized total spectra number, percent coverage and protein identification probability of the proteins identified by mass spectrometry in the bacterial total lysates.

Strain	Description	Accession Number	Gene code	Normalized Total Spectra	Percentage coverage	Protein Identification Probability
E. coli MG1655 + pBAD33-*lolA1*	LolA1	A5FI22	*Fjoh_2111*	90	74%	100%
	LolA	P61316	*lolA*	36	59%	100%
	LolB	P61320	*lolB*	36	70%	100%
E. coli MG1655 + pBAD33-*lolB1*	LolB1	A5FL25	*Fjoh_1066*	159	85%	100%
	LolA	P61316	*lolA*	36	59%	100%
	LolB	P61320	*lolB*	27	62%	100%
E. coli MG1655 + pBAD33-*lolA1lolB1*	LolA1	A5FI22	*Fjoh_2111*	85	74%	100%
	LolB1	A5FL25	*Fjoh_1066*	27	53%	100%
	LolA	P61316	*lolA*	44	57%	100%
	LolB	P61320	*lolB*	25	62%	100%
	LolA	P61316	*lolA*	238	70%	100%

E. coli MG1655 mini λ Tet Δ*lolA* + pBAD33-*lolA*	LolB	P61320	*lolB*	27	67%	100%
E. coli MG1655 mini λ Tet Δ*lolB* + pBAD33-*lolB*	LolA	P61316	*lolA*	25	53%	100%
	LolB	P61320	*lolB*	73	76%	100%
F. johnsoniae UW101 Δ*lolA1* + pCP23-P*ermF-lolA* (*E. coli*)	LolA	P61316	*lolA*	15	63%	100%
	LolA1	A5FI22	*Fjoh_2111*	0	-	-
	LolB1	A5FL25	*Fjoh_1066*	83	63%	100%
F. johnsoniae UW101 Δ*lolB1* + pCP23-P*ermF-lolB* (*E. coli*)	LolB	P61320	*lolB*	43	73%	100%
	LolA1	A5FI22	*Fjoh_2111*	54	75%	100%
	LolB1	A5FL25	*Fjoh_1066*	0	-	-
F. johnsoniae UW101 Δ*lolA1*Δ*lolB1* + pCP23-P*ermF-lolAlolB* (*E. coli*)	LolA	P61316	*lolA*	21	53%	100%
	LolB	P61320	*lolB*	18	55%	100%
	LolA1	A5FI22	*Fjoh_2111*	0	-	-
	LolB1	A5FL25	*Fjoh_1066*	0	-	-

Supplementary Table 4. **LolA and LolB homologs in several Bacteroidota species**.

Homologs identified by DELTA Blast [1] search using LolA1 (WP_081432686.1), LolA2 (WP_012023170.1), LolA3 (WP_012022695.1), LolB1 (WP_012023151.1) and LolB2 (WP_012023169.1) sequences of *F. johnsoniae* as queries (E value ≤ 0.001).

Query	Species	Strain	RefSeq assembly	Protein accession	% ID	E value
LolA1	*Bacteroides fragilis*	NCTC 9343	GCF_000025985.1	WP_005795926.1	20.4	1.11E-27
LolB1	*Bacteroides fragilis*	NCTC 9343	GCF_000025985.1	WP_005784040.1	17.2	7.24E-17
LolA1	*Bacteroides ovatus*	ATCC 8483	GCF_001314995.1	WP_004301819.1	22	1.22E-26
LolA2	*Bacteroides ovatus*	ATCC 8483	GCF_001314995.1	WP_004323602.1	23	2.17E-23
LolB1	*Bacteroides ovatus*	ATCC 8483	GCF_001314995.1	WP_004298193.1	16.5	6.00E-13
LolB2	*Bacteroides ovatus*	ATCC 8483	GCF_001314995.1	WP_004324728.1	23.5	9.80E_8
LolA1	*Bacteroides thetaioataomicron*	DSM 2079	GCF_014131755.1	WP_011109203.1	22.5	2.05E-26
LolB1	*Bacteroides thetaioataomicron*	DSM 2079	GCF_014131755.1	WP_011108844.1	17.3	1.11E-13
LolA1	*Bergeyella zoohelcum*	ATCC 43767	GCF_000301075.1	WP_002663348.1	28.5	2.13E_17
LolB1	*Bergeyella zoohelcum*	ATCC 43767	GCF_000301075.1	WP_002664277.1	21.5	1.18E-29
LolA1	*Capnocytophaga canimorsus*	Cc5	GCF_000220625.1	WP_013997750.1	61.5	7.31E-30
LolB1	*Capnocytophaga canimorsus*	Cc5	GCF_000220625.1	WP_126321423.1	25.6	1.65E-42
LolA1	*Capnocytophaga canis*	CcD38	GCF_000827555.1	WP_042010073.1	57.5	1.3E-30
LolB1	*Capnocytophaga canis*	CcD38	GCF_000827555.1	WP_042009446.1	27.2	1.75E-42
LolA1	*Capnocytophaga cynodegmi*	DSM 19736	GCF_000379185.1	WP_018278573.1	58.4	1.17E-38
LolB1	*Capnocytophaga cynodegmi*	DSM 19736	GCF_000379185.1	WP_018278328.1	29.5	2.79E-55
LolA1	*Capnocytophaga gingivalis*	ATCC 33624	GCF_000174755.1	EEK14233.1 and EEK14289.1 (fragments)	62.5 (EEK14233.1) and 48.9 (EEK14289.1)	4E-15 (EEK14233.1) and 3E-08 (EEK14289.1)
LolA2	*Capnocytophaga gingivalis*	ATCC 33624	GCF_000174755.1	WP_002666178.1	45.6	7.35E-29
LolB1	*Capnocytophaga gingivalis*	ATCC 33624	GCF_000174755.1	WP_040359568.1	25.8	1.68E-34
LolB2	*Capnocytophaga gingivalis*	ATCC 33624	GCF_000174755.1	WP_002666377.1	28.2	9.89E-16

LolA1	*Capnocytophaga ochracea*	DSM 7271	GCF_000023285.1	WP_015782582.1	58.8	1.94E-33
LolA2	*Capnocytophaga ochracea*	DSM 7271	GCF_000023285.1	WP_015782811.1	44.9	9.41E-28
LolB1	*Capnocytophaga ochracea*	DSM 7271	GCF_000023285.1	WP_015782004.1	31.5	1.25E-56
LolB2	*Capnocytophaga ochracea*	DSM 7271	GCF_000023285.1	WP_015782812.1	33.3	1.63E-22
LolA1	*Chitinophaga filiformis*	DSM 527	GCF_900102545.1	WP_089836732.1	24.8	5.14E-36
LolA2	*Chitinophaga filiformis*	DSM 527	GCF_900102545.1	WP_089838851.1	27.4	1.88E-33
LolB1	*Chitinophaga filiformis*	DSM 527	GCF_900102545.1	WP_089834398.1	22	3.14E-43
LolB2	*Chitinophaga filiformis*	DSM 527	GCF_900102545.1	WP_245705585.1	27.4	1.88E-33
LolA1	*Chitinophaga pinensis*	DSM 2588	GCF_000024005.1	WP_012793835.1	27.3	1.25E-36
LolA2	*Chitinophaga pinensis*	DSM 2588	GCF_000024005.1	WP_012789541.1	27.6	5.39E-36
LolB1	*Chitinophaga pinensis*	DSM 2588	GCF_000024005.1	WP_044217960.1	23.4	4.00E-44
LolB2	*Chitinophaga pinensis*	DSM 2588	GCF_000024005.1	WP_012789542.1	28.5	1.08E-17
LolA1	*Gramella forsetii*	KT0803	GCF_000060345.1	WP_011710052.1	54.9	6.98E-35
LolA2	*Gramella forsetii*	KT0803	GCF_000060345.1	WP_011709538.1	38	5.53E-33
LolB1	*Gramella forsetii*	KT0803	GCF_000060345.1	WP_011711102.1)	41.5	4.58E-77
LolA1	*Croceibacter atlanticus*	HTCC2559	GCF_000196315.1	WP_041241137.1	59.6	8.93E-34
LolA2	*Croceibacter atlanticus*	HTCC2559	GCF_000196315.1	WP_013186535.1	48.8	2.09E-32
LolB1	*Croceibacter atlanticus*	HTCC2559	GCF_000196315.1	WP_041240893.1	37.6	1.77E-75
LolB2	*Croceibacter atlanticus*	HTCC2559	GCF_000196315.1	WP_013186534.1	51.2	8.71E-49
LolA1	*Cytophaga hutchinsonii*	ATCC 33406	GCF_000014145.1	WP_011586060.1	30	3.17E-36
LolA2	*Cytophaga hutchinsonii*	ATCC 33406	GCF_000014145.1	WP_011585492.1	30	1.3E-24
LolB1	*Cytophaga hutchinsonii*	ATCC 33406	GCF_000014145.1	WP_041932091.1	26.4	2.04E-50
LolB2	*Cytophaga hutchinsonii*	ATCC 33406	GCF_000014145.1	WP_011585491.1	31.4	9.43E-15
LolA1	*Flavobacterium columnare*	NBRC 100251	GCF_007990835.1	WP_077225215.1	59.6	8.85E-34
LolA2	*Flavobacterium columnare*	NBRC 100251	GCF_007990835.1	WP_014166421.1	59.6	8.31E-33
LolA3	*Flavobacterium columnare*	NBRC 100251	GCF_007990835.1	WP_097609575.1	37.9	4.4E-34
LolB1	*Flavobacterium columnare*	NBRC 100251	GCF_007990835.1	WP_097609630.1	41.3	2.78E-58
LolB2	*Flavobacterium columnare*	NBRC 100251	GCF_007990835.1	WP_041253277.1	39.6	1.12E-36
LolA1	*Flavobacterium johnsoniae*	ATCC 17061	GCF_034479105.1	WP_081432686.1	100	2.37E-41

LolA2	*Flavobacterium johnsoniae*	ATCC 17061	GCF_034479105.1	WP_012023170.1	100	4.76E-135
LolA3	*Flavobacterium johnsoniae*	ATCC 17061	GCF_034479105.1	WP_012022695.1	100	1.78E-138
LolB1	*Flavobacterium johnsoniae*	ATCC 17061	GCF_034479105.1	WP_012023151.1	100	1.78E-104
LolB2	*Flavobacterium johnsoniae*	ATCC 17061	GCF_034479105.1	WP_012023169.1	100	4.76E-135
LolA1	*Elizabethkingia meningoseptica*	NBRC 12535	GCF_000367325.1	WP_019051346.1	27.1	8.2E-21
LolB1	*Elizabethkingia meningoseptica*	NBRC 12535	GCF_000367325.1	WP_026149339.1	26.8	1.43E-39
LolA1	*Flavobacterium psychrophilum*	DSM 3660	GCF_900101925.1	WP_011963703.1	64.8	5.64E-36
LolA2	*Flavobacterium psychrophilum*	DSM 3660	GCF_900101925.1	WP_011964352.1	75.2	4.85E-35
LolB1	*Flavobacterium psychrophilum*	DSM 3660	GCF_900101925.1	WP_011964528.1	43.3	1.2E-67
LolB2	*Flavobacterium psychrophilum*	DSM 3660	GCF_900101925.1	WP_034099413.1	64.6	2.73E-78
LolA1	*Flavobacterium succinicans*	LMG 10402	GCF_000611675.1	WP_024980104.1	82.7	3.01E-38
LolB1	*Flavobacterium succinicans*	LMG 10402	GCF_000611675.1	WP_024980583.1	56.8	1.28E-70
LolA1	*Flexibacter flexilis*	NBRC 15060	GCF_900112255.1	WP_091512919.1	17.8	1.89E-28
LolB1	*Flexibacter flexilis*	NBRC 15060	GCF_900112255.1	WP_221405328.1	22.2	4.81E-27
LolA1	*Kordia algicida*	OT-1	GCF_000154725.1	WP_040559877.1	54.2	1.98E-35
LolA3	*Kordia algicida*	OT-1	GCF_000154725.1	WP_007093428.1	21.3	1.58E-8
LolB1	*Kordia algicida*	OT-1	GCF_000154725.1	WP_238528710.1	35.1	5.24E-63
LolA1	*Polaribacter irgensii*	23-P	GCF_000153225.1	WP_004570735.1	31	3.78E-24
LolB1	*Polaribacter irgensii*	23-P	GCF_000153225.1	WP_004569654.1	29.6	5.84E-53
LolA1	*Porphyromonas gingivalis*	ATCC 33277	GCF_000010505.1	WP_012457548.1	20.7	2.13E-14
LolA3	*Porphyromonas gingivalis*	ATCC 33277	GCF_000010505.1	WP_004584472.1	25	5.45E-12
LolB1	*Porphyromonas gingivalis*	ATCC 33277	GCF_000010505.1	WP_012457941.1	17.9	1.05E-35
LolA1	*Prevotella intermedia*	ATCC 25611	GCF_000439065.1	WP_028905298.1	16.3	1.48E-20
LolA3	*Prevotella intermedia*	ATCC 25611	GCF_000439065.1	WP_004367589.1	18.6	4.73E-11

LolB1	*Prevotella intermedia*	ATCC 25611	GCF_000439065.1	WP_028905940.1	12.6	1.16E-28
LolA1	*Prevotella melaninogenica*	ATCC 25845	GCF_000144405.1	WP_013265419.1	17.8	9.11E-22
LolB1	*Prevotella melaninogenica*	ATCC 25845	GCF_000144405.1	WP_013264029.1	15.2	6.31E-29
LolA1	*Riemerella anatipestifer*	ATCC 11845	GCF_000183155.1	WP_004919104.1	31	2.35E-18
LolB1	*Riemerella anatipestifer*	ATCC 11845	GCF_000183155.1	WP_004916613.1	25.5	4.92E-31
LolA1	*Sphingobacterium mizutaii*	NBRC 14946	GCF_007990895.1	WP_093100220.1	27.9	1.91E-27
LolB1	*Sphingobacterium mizutaii*	NBRC 14946	GCF_007990895.1	WP_236736514.1	24.7	1.15E-42
LolA1	*Sporocytophaga myxococcoides*	DSM 11118	GCF_000426725.1	WP_028982018.1	26.6	1.72E-41
LolA2	*Sporocytophaga myxococcoides*	DSM 11118	GCF_000426725.1	WP_051312992.1	32.4	7.73E-36
LolA3	*Sporocytophaga myxococcoides*	DSM 11118	GCF_000426725.1	WP_028981309.1	21.3	1.16E-9
LolB1	*Sporocytophaga myxococcoides*	DSM 11118	GCF_000426725.1	WP_028981195.1	25.4	3.04E-34
LolB2	*Sporocytophaga myxococcoides*	DSM 11118	GCF_000426725.1	WP_028979568.1	34.6	9.67E-24
LolA1	*Xanthomarina gelatinilytica*	AK20	GCF_000348685.1	WP_007646658.1	59.1	4.35E-39
LolA2	*Xanthomarina gelatinilytica*	AK20	GCF_000348685.1	WP_007647468.1	48.2	2.63E-29
LolB1	*Xanthomarina gelatinilytica*	AK20	GCF_000348685.1	WP_007647364.1	30.4	8.9E-53
LolB2	*Xanthomarina gelatinilytica*	AK20	GCF_000348685.1	WP_007647467.1	40.6	1.66E-35
LolA1	*Zobellia galactanivorans*	DsiJT	GCF_000973105.1	WP_013995938.1	55.7	1.05E-33
LolA2	*Zobellia galactanivorans*	DsiJT	GCF_000973105.1	WP_013993418.1	42.4	1.58E-29
LolB1	*Zobellia galactanivorans*	DsiJT	GCF_000973105.1	WP_013993148.1	35.1	7.35E-66
LolB2	*Zobellia galactanivorans*	DsiJT	GCF_000973105.1	WP_013993417.1	36.5	4.1E-38

Supplementary Table 5. Gliding and T9SS-related proteins detected in the OM of the *lolA1* and *lolB1* mutants.
Fold change and significance values between brackets are respectively < 1.5 and < 20.

Description	Signal peptide	Accession number	Gene code	Δ*lolA1*/WT FC	Significance	Δ*lolB1*/WT FC	Significance
SprD	SPI	A1E5U4	*Fjoh_0980*	0.05	28.23	0.47	(17.00)
GldJ	SPII	A5FJM9	*Fjoh_1557*	0.05	51.16	0.22	51.32
SprE	SPII	A1E5T9	*Fjoh_1051*	0.05	(7.03)	0.06	(7.90)
SprF-like protein	SPI	A5FE72	*Fjoh_3477*	0.15	35.59	0.48	(16.91)
SprF-like protein	SPI	A5FCV6	*Fjoh_3951*	0.16	49.23	0.16	47.24
SprF	SPI	A5FLA7	*Fjoh_0978*	0.37	28.54	(0.84)	(1.22)
SprT	SPI	A5FJX0	*Fjoh_1466*	0.41	29.85	1.67	(18.94)
GldK	SPII	A5FIS8	*Fjoh_1853*	0.48	30.84	0.08	59.60
GldN	SPI	A5FIT1	*Fjoh_1856*	0.53	25.61	0.33	52.46
SprB	SPI	A1E5U5	*Fjoh_0979*	(0.78)	(12.22)	0.32	47.70
PorV	SPI	A5FJM7	*Fjoh_1555*	(0.80)	(10.08)	(1.35)	22.91
SprA	SPI	Q5I6C7	*Fjoh_1653*	(0.81)	(7.74)	(0.80)	(8.12)
GldM	SPI	A5FIT0	*Fjoh_1855*	(0.92)	(3.92)	(0.78)	26.20
SprF-like protein	SPI	A5FJB8	*Fjoh_1677*	(0.95)	(0.74)	1.85	27.43
RemH	SPI	A5FL98	*Fjoh_0984*	(1.01)	(0.71)	1.78	21.32
GldB	SPII	A5FJ02	*Fjoh_1793*	(1.08)	(1.08)	(0.90)	(9.54)
RemF	SPI	A5FEZ9	*Fjoh_3206*	(1.12)	(0.65)	1.93	20.40
RemG	SPI	A5FL97	*Fjoh_0983*	(1.22)	(5.03)	(1.23)	(10.18)
GldI	SPII	A5FHC1	*Fjoh_2369*	(1.43)	30.45	(1.02)	(0.99)
GldH	SPII	Q8KRP0	*Fjoh_0890*	2.31	39.28	(1.23)	(12.76)
PorU	SPI	A5FJM8	*Fjoh_1556*	nd	-	1.51	33.50
RemI	SPI	A5FF07	*Fjoh_3194*	2.72	23.89	1.66	23.81
GldD	SPII	A5FJP0	*Fjoh_1540*	nd	-	nd	-

Supplementary Table 6. Bacterial strains used in this study.

Strain	Genotype and/or description	Reference
Flavobacterium johnsoniae		
WT	*F. johnsoniae* UW101	[2]
ΔgldJ	Deletion of *Fjoh_1557*	This study and [3]
ΔgldJ-548	Deletion of *Fjoh_1557* residues 549-561	This study and [4]
ΔlolA1	Deletion of *Fjoh_2111*	This study
ΔlolA2	Deletion of *Fjoh_1085*	This study
ΔlolB1	Deletion of *Fjoh_1066*	This study
ΔlolB2	Deletion of *Fjoh_1084*	This study
ΔlolA3	Deletion of *Fjoh_0605*	This study
ΔlolA1ΔlolB1	Deletion of *Fjoh_2111* and *Fjoh_1066*	This study
ΔlolA2ΔlolB2	Deletion of *Fjoh_1084-1085*	This study
ΔlolA1ΔlolA2 ΔlolA3ΔlolB1ΔlolB2	Deletion of *Fjoh_2111, Fjoh_1066*, *Fjoh_1084-1085*, and *Fjoh_0605*	This study
Escherichia coli		
Top10	F-mcrAΔ(mrr-hsdRMS-mcrBC) φ80lacZΔM15ΔlacX74 recA1araD139Δ(araleu)7697 galU galK rpsL endA1 nupG; Sm^R	Invitrogen
MT607	*pro-82 thi-I hsdR17 (r-m+) supE44 recA56*	Received from R. Hallez lab and [5]
MG1655 mini-λ-Tet	MG1655 *mini-λ-Tet*	[6]
Capnocytophaga canimorsus		
Cc5	Wild type (BCCM-LMG 28512)	[7]

Supplementary Table 7. Plasmids used in this study.

Description	Reference	
**Vectors**		
pYT354	Suicide vector carrying *sacB*; MCS of pBC SK+ cloned into pYT313; Amp^{R} (Ery^{R})	[8]
pCP23	ColE1 ori; (pCP1 ori); Amp^{R} (Tet^{R});*E. coli-F. johnsoniae* shuttle plasmid	[9]
pCP23-P*ermF*	pCP23 with P*ermF* promoter and MCS from pMM47.A cloned into BamHI and PstI restriction sites	This study
pBAD33	p15A *ori*; Cm^{R}. Low copy *E. coli* expression plasmid with arabinose inducible promoter	[10]
pMM47.A	ColE1 *ori*; (pCC7 *ori*); Amp^{R}; (Cfx^{R}). *E. coli-C. canimorsus* expression shuttle plasmid with *ermF* promoter	[7]
pKD4	Template plasmid for gene disruption in *E. coli* with the Kan^{R} gene flanked by FRT sites	Received from R. Hallez lab and [11]
**Suicide plasmids**		
pYT313-*gldJ*-KO	Deletion of *Fjoh_1557.*	Received from Ben Berks' lab
pYT313-*gldJ-548*	Deletion of amino acids 548-561 of *Fjoh_1557.*	Received from Ben Berks' lab
pYT354-*lolA1*-KO	Deletion of *Fjoh_2111.* Upstream and downstream regions of *Fjoh_2111* amplified with oligonucleotides 8577 and 8503 and 8504 and 8578 respectively from gDNA and cloned sequentially into pYT354 using ApaI, XhoI and SpeI restriction sites.	This study
pYT354-*lolA2*-KO	Deletion of *Fjoh_1085.* Upstream and downstream regions of *Fjoh_1085* amplified with oligonucleotides 8593 and 8594 and 8595 and 8596 respectively from gDNA and cloned sequentially into pYT354 using SphI, XhoI, and SpeI restriction sites.	This study
pYT354-*lolA3*-KO	Deletion of *Fjoh_0605.* Upstream and downstream regions of *Fjoh_0605* amplified with oligonucleotides TD3 and TD4 and TD5 and TD6 respectively from gDNA and cloned into pYT354 (amplified with oligonucleotides TD1 and TD2) by Gibson assembly.	This study
pYT354-*lolB1*-KO	Deletion of *Fjoh_1066.* Upstream and downstream regions of *Fjoh_1066* amplified with oligonucleotides 8619 and 8620 and 8621 and 8622 respectively from gDNA and cloned sequentially into pYT354 using SphI, XhoI and SpeI restriction sites.	This study
pYT354-*lolB2*-KO	Deletion of *Fjoh_1084.* Upstream and downstream regions of *Fjoh_1084* amplified with oligonucleotides 8625 and 8626 and 8627 and 8628 respectively from gDNA and cloned sequentially into pYT354 using SphI, XhoI and BamHI restriction sites.	This study

pYT354-*lolA2-lolB2*-KO	Deletion of *Fjoh_1084* and *Fjoh_1085.* Upstream region of *Fjoh_1085* and downstream region of *Fjoh_1084* amplified with oligonucleotides 8593 and 8594 and 8627 and 8628 respectively from gDNA and cloned sequentially into pYT354 using SphI, XhoI and BamHI restriction sites.	This study
pYT354-*Ccan_17050*-KO	Deletion of *C. canimorsus Ccan_17050* (*lolB*). Upstream and downstream regions of *Ccan_17050* amplified with oligonucleotides 8631 and 8632 and 8633 and 8634 respectively from gDNA. The two fragments were fused by PCR using oligonucleotides 8631 and 8634 and cloned into pYT354 using SphI and XhoI restriction sites.	This study
**Expression plasmids**		
pCP23-P*ermF*-*siaC*	WT sialidase cloned into pCP23-P*ermF*.	This study
pCP23-P*ermF*-LES-*siaC*	Sialidase harboring the *F. johnsoniae* LES sequence (SDDFE) cloned into pCP23-P*ermF*.	This study
pCP23-P*ermF*-*lolA1*	Full length *Fjoh_2111* amplified with oligonucleotides 8499 and 8500 and cloned into pCP23-P*ermF* using NcoI and XhoI restriction sites.	This study
pCP23-P*ermF*-*lolB1*	Full length *Fjoh_1066* amplified with oligonucleotides 8635 and 8636 and cloned into pCP23-P*ermF* using NcoI and XhoI restriction sites.	This study
pCP23-P*ermF*-*lolA1*-P*ermF*-*lolB1*	Full length *Fjoh_1066* amplified with *ermF* promoter from pCP23-P*ermF*-*lolB1* with oligonucleotides 8644 and 8645 and cloned into pCP23-P*ermF*-*lolA1* using XhoI and SpeI restriction sites.	This study
pCP23-P*ermF*-*lolA* *(E. coli)*	Full length *lolA* amplified from *E. coli* gDNA with oligonucleotides 8591 and 8592 and cloned into pCP23-P*ermF* using NcoI and XbaI restriction sites.	This study

pCP23-P*ermF*-*lolB* (*E. coli*)	Full length *lolB* amplified *from E. coli* gDNA with oligonucleotides 5434 and 5435 and cloned into pCP23-P*ermF* using NcoI and XbaI restriction sites.	This study
pCP23-P*ermF*-*lolA*-*lolB* (*E. coli*)	Full length *lolB* amplified from *E. coli* gDNA with oligonucleotides 8638 and 8639 and cloned into pCP23-P*ermF*-*lolA* (*E. coli*) using XbaI and SpeI restriction sites.	This study
pCP23-P*ermF*-*lolA* (*C. canimorsus*)	Full length *lolA* (*Ccan_16490*) amplified from *C. canimorsus* Cc5 gDNA with primers 7203 and 7204 and cloned into pCP23-P*ermF* using NcoI and XbaI restriction sites.	This study
pCP23-P*ermF*-*lolB* (*C. canimorsus*)	Full length *lolB* (*Ccan_17050*) amplified from *C. canimorsus* Cc5 gDNA with primers 8687 and 8688 and cloned into pCP23-P*ermF* using NcoI and XhoI restriction sites.	This study
pCP23-P*ermF*-*lolB1L74E-His*	First fragment of *Fjoh_1066* was amplified with primers 8635 and TD12 while second fragment was amplified with TD11 and 8850 (no stop codon). The two fragments were fused by PCR using primers 8635 and 8850 and cloned into pCP23-P*ermF* using NcoI and XhoI restriction sites.	This study
pCP23-P*ermF*-*lolB1Δ73-76-His*	First fragment of *Fjoh_1066* was amplified with primers 8635 and TD18 while second fragment was amplified with TD17 and 8850 (no stop codon). The two fragments were fused using primers 8635 and 8850 and cloned into pCP23-P*ermF* using NcoI and XhoI restriction sites.	This study
pCP23-P*ermF*-*lolB1Δ223-243-His*	*Fjoh_1066* was amplified with primers 8635 and TD25 (no stop codon) and cloned into pCP23-P*ermF* using NcoI and XhoI restriction sites.	This study
pCP23-P*ermF*-*mlolB1*	Full length *Fjoh_1066* with C17G mutation amplified with primers 8643 and 8688 and cloned into pCP23-P*ermF* using NcoI and XhoI restriction sites.	This study
pBAD33-*lolA*	Full length *lolA* with its RBS amplified from *E. coli* gDNA with primers 8660 and 8592 and cloned into pBAD33 using KpnI and XbaI restriction sites.	This study

pBAD33-*lolB*	Full length *lolB* with RBS amplified from *E. coli* gDNA with primers 8661 and 5435 and cloned into pBAD33 using KpnI and XbaI restriction sites.	This study
pBAD33-*lolA1*	Full length *Fjoh_2111* amplified with primers 8663 and 8651 and cloned into pBAD33 using KpnI and XbaI restriction sites.	This study
pBAD33-*lolA2*	Full length *Fjoh_1085* amplified with primers 8665 and 8655 and cloned into pBAD33 KpnI and XbaI restriction sites.	This study
pBAD33-*lolB1*	Full length *Fjoh_1066* amplified with oligonucleotides 8664 and 8653 and cloned into pBAD33 using KpnI and XbaI restriction sites.	This study
pBAD33-*lolB2*	Full length *Fjoh_1084* amplified with primers 8666 and 8657 and cloned into pBAD33 using KpnI and XbaI restriction sites.	This study
pBAD33-*lolA1lolB1*	Full length *Fjoh_1066* amplified with primers 8667 and 8659 and cloned into pBAD33-*lolA1* using XbaI and SphI restriction sites.	This study
pBAD33-*lolA2lolB2*	Full length *Fjoh_1084-85* amplified with primers 8666 and 8655 and cloned into pBAD33 using KpnI and XbaI restriction sites.	This study
pMM47-*lolA1*	Full length *Fjoh_2111* amplified with oligonucleotides 8499 and 8500 and cloned into pMM47.A using NcoI and XhoI restriction sites.	This study
pMM47-*lolB1*	Full length *Fjoh_1066* amplified with oligonucleotides 8635 and 8636 and cloned into pMM47.A using NcoI and XhoI restriction sites.	This study

pFL63	pFL63 expressing *C. canimorsus lolA Ccan_16490*; Tet^R	This study

Supplementary Table 8. Oligonucleotides used in this study.

Name	Sequence 5'-3'
5434	CATGCCATGGGACCCCTGCCCGATTTT
5435	GCTCTAGATTATTTCACTATCCAGTTATCC
7203	CCCCATGGGGAAAAAGATACTATTGTTAATATC
7204	GGTCTAGATTATAGTTCTGAAATATAGTATCC
8499	CATACCATGGGAAACAAAATTAATCCAATCATG
8500	CCGCTCGAGTTAATCTAATTTATTGATGTAG
8504	CCGCTCGAGGTGCCGACTACAGGAAAAGAC
8578	TGCACTACTAGTAGCAGCAGAAATACCAGTTG
8591	CATGCCATGGGAAAAAAAATTGCCATCACCTGT
8592	GCTCTAGACTACTTACGTTGATCATCTACCG
8593	CATGCATGCTATCTAATTCTTTCGGATTTGGAGG
8594	CCGCTCGAGGTGCTTAATGTTTTTCCCAC
8595	CCGCTCGAGCGACAAAGAATTTACGATTTCG
8596	GCACTACTAGTATAGTAATCGCTGTCCAGAATATC
8619	CATGCATGCGAACAGCCTGAAGCTTTTGG
8620	CCGCTCGAGCTTAATTTCTCCTTACTTCTTTTG
8621	CCGCTCGAGTACCAAGCGGTTATAAAAAAG
8622	GCACTACTAGTCTCACTTCACCTTGCAGAATATC
8625	CATGCATGCGCGGTGCCGTTTATGGTGAAG
8626	CCGCTCGAGCCAAAACGATTGCCAGAAAGC
8627	CCGCTCGAGCAAGTCTGAGTAGAATATTAAAC
8628	CGGGATCCGTAACCCGTAATAGCCGGAAC
8631	CATGCATGCAATGTTGATGCTCGTGATGG
8632	ACGGAATACGGTACGGTGCGGTGTATTTAACTGATGGTAA

8633	TTAAATACACCGCACCGTACCGTATTCCGTCGGG
8634	CCGCTCGAGATTGCTTTATGTAAAGCATACG
8635	CATACCATGGGAAAAAAATATATTATAATAG
8636	CCGCTCGAGTTACTTAATTAAAACTTTTTTATAAC
8638	GCTCTAGAATGCCCCTGCCCGATTTTCGTC
8639	GCACTACTAGTTTATTTCACTATCCAGTTATCC
8643	CATACCATGGGAAAAAAATATATTATAATAGTATTAATATCGGTTTTTGTGGTTTCAGGTAAATC
8651	GGGGTACCATGAAAAAATATATTATAATAG
8653	GCTCTAGATTACTTAATTAAAACTTTTTTATAAC
8655	GCTCTAGATTAATTAGTAAAAACTGAATC
8657	GCTCTAGACTACTCAGACTTGAAATAATTC
8659	ACATGCATGCTTACTTAATTAAAACTTTTTTATAAC
8660	GGGTACCCGGGAGTGACGTAATTTGAG
8661	GGGGTACCAGGGTTATAACTGCAACGTATC
8663	GGGGTACCAGGAGGACAGCTATGAACAAAATTAATCCAATC
8664	GGGGTACCAGGAGGACAGCTATGAAAAAATATATTATAATAG
8665	GGGGTACCAGGAGGACAGCTATGAAAACTAAAATAGCTCTAC
8666	GGGGTACCAGGAGGACAGCTATGCAAAAATCGACGATTCAG
8667	GCTCTAGAAGGAGGACAGCTATGAAAAAATATATTATAATAG
8675	ATTATTAGCCTGGAATAGAGAGTAGAGGGAACTCCCGATGTGTAGGCTGGAGCTGCTTC
8677	CTTGAACATAGACGATAGCGGACGGTAACGCTAGCATTAGTGTAGGCTGGAGCTGCTTC
8679	TCCGAAAAATCGAGCGACAGATTGCTCACTCAGGTGCCTCATATGAATATCCTCCTTA
8680	CAGATTAAGTTTTGCCGGAGAGGGCCACTGTGTCCGCATCATATGAATATCCTCCTTA
8687	CATACCATGGGAAAATACCTTTTTCTGAAAATAC
8688	CCGCTCGAGTTAATTATTGATTGCTTTCG
8850	CCGCTCGAGCTTAATTAAAACTTTTTTATAACCGC
TD1	GAATTGAAGAAGACGGTATTCGGTATCGATAAGCTTGATATCGAATTCCTGCAGCCC
TD2	TATCTTTCTGTTACGGTTATTTCTTTTGTAAATGTCGACCTCGAGGGGGGGCC
TD3	GGCCCCCCCTCGAGGTCGACATTTACAAAAGAAATAACCGTAACAGAAAGATATT
TD4	TCTCGGCCGGAACAATTTGTTTTTTAGCCGATGCTTTTATGGTGTTGTATTTGTC
TD5	GACAAATACAACACCATAAAAGCATCGGCTAAAAAACAAATTGTTCCGGCCGAGA
TD6	GGGCTGCAGGAATTCGATATCAAGCTTATCGATACCGAATACCGTCTTCTTCAATTC
TD11	ACAGATTTTAATAAGCGTTAGATTCGAGGGAATTACAATGGCAAAAGCTTTAA
TD12	TTAAAGCTTTTGCCATTGTAATTCCCTCGAATCTAACGCTTATTAAAATCTGT

TD17	CAAACAGATTTTAATAAGCGTTAGAACAATGGCAAAAGCTTTAATAACAC
TD18	GTGTTATTAAAGCTTTTGCCATTGTTCTAACGCTTATTAAAATCTGTTTG
TD25	CCGCTCGAGTGAAATGTTATTGTAATTCAGATTA

Supplementary References

1 Boratyn, G. M. *et al.* Domain enhanced lookup time accelerated BLAST. *Biol Direct* **7**, 12 (2012). https://doi.org:10.1186/1745-6150-7-12

2 McBride, M. J. *et al.* Novel features of the polysaccharide-digesting gliding bacterium Flavobacterium johnsoniae as revealed by genome sequence analysis. *Appl Environ Microbiol* **75**, 6864-6875 (2009). https://doi.org:10.1128/AEM.01495-09

3 Braun, T. F. & McBride, M. J. Flavobacterium johnsoniae GldJ is a lipoprotein that is required for gliding motility. *J Bacteriol* **187**, 2628-2637 (2005). https://doi.org:10.1128/JB.187.8.2628-2637.2005

4 Johnston, J. J., Shrivastava, A. & McBride, M. J. Untangling Flavobacterium johnsoniae Gliding Motility and Protein Secretion. *J Bacteriol* **200** (2018). https://doi.org:10.1128/JB.00362-17

5 Aneja, P. & Charles, T. C. Poly-3-hydroxybutyrate degradation in Rhizobium (Sinorhizobium) meliloti: isolation and characterization of a gene encoding 3-hydroxybutyrate dehydrogenase. *J Bacteriol* **181**, 849-857 (1999). https://doi.org:10.1128/JB.181.3.849-857.1999

6 Court, D. L. *et al.* Mini-lambda: a tractable system for chromosome and BAC engineering. *Gene* **315**, 63-69 (2003). https://doi.org:10.1016/s0378-1119(03)00728-5

7 Mally, M. & Cornelis, G. R. Genetic tools for studying Capnocytophaga canimorsus. *Appl Environ Microbiol* **74**, 6369-6377 (2008). https://doi.org:10.1128/AEM.01218-08

8 Zhu, Y. *et al.* Genetic analyses unravel the crucial role of a horizontally acquired alginate lyase for brown algal biomass degradation by Zobellia galactanivorans. *Environ Microbiol* **19**, 2164-2181 (2017). https://doi.org:10.1111/1462-2920.13699

9 Agarwal, S., Hunnicutt, D. W. & McBride, M. J. Cloning and characterization of the Flavobacterium johnsoniae (Cytophaga johnsonae) gliding motility gene, gldA. *Proc Natl Acad Sci U S A* **94**, 12139-12144 (1997). https://doi.org:10.1073/pnas.94.22.12139

10 Guzman, L. M., Weiss, D. S. & Beckwith, J. Domain-swapping analysis of FtsI, FtsL, and FtsQ, bitopic membrane proteins essential for cell division in Escherichia coli. *J Bacteriol* **179**, 5094-5103 (1997). https://doi.org:10.1128/jb.179.16.5094-5103.1997

11 Datsenko, K. A. & Wanner, B. L. One-step inactivation of chromosomal genes in Escherichia coli K-12 using PCR products. *Proc Natl Acad Sci U S A* **97**, 6640-6645 (2000). https://doi.org:10.1073/pnas.120163297
