# Supplementary material for: LolA and LolB are conserved in Bacteroidota and are crucial for gliding motility and Type IX secretion
Source: Commun Biol. 2025 Mar 6;8:376. doi: 10.1038/s42003-025-07817-2 (PMC11885536; doi:10.1038/s42003-025-07817-2)
Supplement: Supplementary file 3 — Description of Additional Supplementary File [file 42003_2025_7817_MOESM3_ESM.pdf]

Description Of Additional Supplementary File

File name: Supplementary Data 1
Description: Proteins with a SPI/SPII/SPII-LES detected in the OM of the lolA1 and lolB1 mutants (FC ≥ 1.5, significance ≥ 20). Stars (*) indicate proteins for which only a PUL prediction by CAZy was available. Class descriptions are from the EggNOG database 5.0, except for Polysaccharide utilization (which groups all Sus-like proteins and proteins predicted by EggNOG to be involved in carbohydrate transport and metabolism) and Gliding/T9SS, which are custom classes.

File name: Supplementary Data 2
Description: Cytoplasmic and IM proteins detected in the OM of the lolA1 and lolB1 mutants (FC ≥ 1.5, significance ≥ 20).

File name: Supplementary Data 3
Description: Numerical source data of graphs and charts of Figs. 2c, 4a, 5a, 5c, 6c, 7c, 9b and 9c. File name: Supplementary Movie 1 Description: Gliding on agar pads of WT, lolA1, lolB1 and gldJ F. johnsoniae mutants.

File name: Supplementary Movie 1
Description: Gliding on agar pads of WT, lolA1, lolB1 and gldJ F. johnsoniae mutants.
