# Supplementary material for: LolA and LolB are conserved in Bacteroidota and are crucial for gliding motility and Type IX secretion
Source: Commun Biol. 2025 Mar 6;8:376. doi: 10.1038/s42003-025-07817-2 (PMC11885536; doi:10.1038/s42003-025-07817-2)
Supplement: Supplementary file 4 — Supplementary Data 1 [file 42003_2025_7817_MOESM4_ESM.docx]

**Supplementary Data 1. Proteins with a SPI/SPII/SPII-LES detected in the OM of the *lolA1* and *lolB1* mutants (FC ≥ 1.5, significance ≥ 20).**

Stars (*) indicate proteins for which only a PUL prediction by CAZy was available.

Class descriptions are from the EggNOG database 5.0, except for Polysaccharide utilization (which groups all Sus-like proteins and proteins predicted by EggNOG to be involved in carbohydrate transport and metabolism) and Gliding/T9SS, which are custom classes.

**Δ*lolA1***

| Accession | Signal peptide | Significance | *lolA1*/WT FC | Description | Gene code | COG Class Description | Localization | CAZy family | PUL (CAZy literature and prediction) | Essentiality |  |  |
| --- | --- | --- | --- | --- | --- | --- | --- | --- | --- | --- | --- | --- |
| A5FIT9 | SPII | 63.91 | 64 | Lipopolysaccharide assembly protein | *Fjoh_1847* | Function unknown | OM lipoprotein |  |  | Yes |  |  |
| A5FMM3 | SPII | 101.76 | 64 | Glycine zipper family protein | *Fjoh_0505* | Function unknown | OM lipoprotein |  |  |  |  |  |
| A5FAM8 | SPI | 41.45 | 64 | SprF-like protein | *Fjoh_4749* | Gliding/T9SS | OM integral protein |  |  |  |  |  |
| A5FHF7 | SPII | 53.13 | 51.03 | Hypothetical lipoprotein | *Fjoh_2342* | Function unknown | OM lipoprotein |  |  |  |  |  |
| A5FNR2 | SPI | 20.18 | 23.99 | OMP_b-brl_2 domain-containing protein | *Fjoh_0127* | Cell wall/membrane/envelope biogenesis | OM integral protein |  |  |  |  |  |
| A5FAI4 | SPII | 69.72 | 20.35 | DUF3300 domain-containing protein | *Fjoh_4779* | RNA processing and modification | OM lipoprotein |  |  |  |  |  |
| A5FAC1 | SPII-LES | 55.28 | 15.71 | Lipocalin-like domain-containing protein | *Fjoh_4843* | Lipid transport and metabolism | Surface-exposed lipoprotein |  |  |  |  |  |
| A5FNT5 | SPII | 45.14 | 14.21 | Hypothetical lipoprotein | *Fjoh_0097* | Function unknown | OM lipoprotein |  |  |  |  |  |
| A5FP10 | SPI | 44.7 | 12.72 | Peptidase, subfamily S1B unassigned peptidases | *Fjoh_0019* | Posttranslational modification, protein turnover, chaperones | Periplasmic |  |  |  |  |  |
| A5FFN3 | SPI | 58.28 | 11.6 | Uncharacterized protein | *Fjoh_2959* | Function unknown | Periplasmic |  |  |  |  |  |
| A5FDE8 | SPI | 36.7 | 7.76 | DUF3570 domain-containing protein | *Fjoh_3759* | Lipid transport and metabolism | OM integral protein |  |  |  |  |  |
| A5FMH4 | SPII-LES | 43.3 | 5.38 | Hypothetical lipoprotein | *Fjoh_0561* | Function unknown | Surface-exposed lipoprotein |  |  |  |  |  |
| A5FBR6 | SPII | 29.15 | 5.11 | DUF4369 domain-containing protein | *Fjoh_4343* | Energy production and conversion | OM lipoprotein |  |  |  |  |  |
| A5FHZ5 | SPII | 37.78 | 4.51 | Hypothetical lipoprotein | *Fjoh_2151* | Function unknown | OM lipoprotein |  |  |  |  |  |
| A5FBX5 | SPII | 50.92 | 4.22 | Efflux transporter, RND family, MFP subunit | *Fjoh_4294* | Cell wall/membrane/envelope biogenesis | IM lipoprotein |  |  |  |  |  |
| A5FM15 | SPI | 50.7 | 4.18 | BatE-like protein | *Fjoh_0723* | Signal transduction mechanisms | Periplasmic |  |  | Yes |  |  |
| A5F9V9 | SPII-LES | 50.24 | 4.14 | Cytochrome-c peroxidase | *Fjoh_5007* | Energy production and conversion | Surface-exposed lipoprotein |  |  | Yes |  |  |
| A5FJP1 | SPII | 42.8 | 3.83 | Heavy metal transport/detoxification protein | *Fjoh_1541* | Inorganic ion transport and metabolism | OM lipoprotein |  |  | Yes |  |  |
| A5FK44 | SPII-LES | 45.57 | 3.81 | Hypothetical lipoprotein | *Fjoh_1393* | Function unknown | Surface-exposed lipoprotein |  |  |  |  |  |
| A5FK25 | SPII | 63.26 | 3.76 | PKD domain containing protein | *Fjoh_1410* | Signal transduction mechanisms | OM lipoprotein |  |  |  |  |  |
| A5FM13 | SPI | 34.37 | 3.6 | BatC-like protein | *Fjoh_0721* | Coenzyme transport and metabolism | Periplasmic |  |  | Yes |  |  |
| A5FMJ3 | SPII-LES | 27.98 | 3.56 | Hypothetical lipoprotein | *Fjoh_0546* | Gliding/T9SS | Surface-exposed lipoprotein |  |  |  |  |  |
| A5FB40 | SPI | 28.82 | 3.3 | Outer membrane protein beta-barrel domain-containing protein | *Fjoh_4578* | Cell wall/membrane/envelope biogenesis | OM integral protein |  |  |  |  |  |
| A5FNB1 | SPII | 41.06 | 3.27 | Peptide-methionine (R)-S-oxide reductase | *Fjoh_0270* | Posttranslational modification, protein turnover, chaperones | OM lipoprotein |  |  |  |  |  |
| A5FCD4 | SPII | 26.92 | 3.15 | Efflux transporter, RND family, MFP subunit | *Fjoh_4132* | Cell wall/membrane/envelope biogenesis | IM lipoprotein |  |  |  |  |  |
| A5FHB6 | SPI | 23.72 | 3.12 | Peptidoglycan-binding LysM | *Fjoh_2379* | Cell wall/membrane/envelope biogenesis | Periplasmic |  |  |  |  |  |
| A5FEP9 | SPII | 32.13 | 3.1 | Glucose sorbosone dehydrogenase | *Fjoh_3307* | Carbohydrate transport and metabolism | OM lipoprotein |  |  |  |  |  |
| A5FF07 | SPI | 23.89 | 2.72 | Gliding motility protein RemI | *Fjoh_3194* | Gliding/T9SS | Cell surface/extracellular |  |  |  |  |  |
| A5FA29 | SPI | 39.82 | 2.68 | Membrane protein involved in aromatic hydrocarbon degradation | *Fjoh_4941* | Lipid transport and metabolism | OM integral protein |  |  |  |  |  |
| A5F9X2 | SPII | 21.3 | 2.64 | DUF6565 domain-containing protein | *Fjoh_5000* | Function unknown | OM lipoprotein |  |  |  |  |  |
| A5FIS7 | SPI | 29.78 | 2.38 | Exopolyphosphatase-like protein | *Fjoh_1868* | Nucleotide transport and metabolism | Periplasmic |  |  |  |  |  |
| A5FBC4 | SPI | 26.05 | 2.37 | DUF541 domain-containing protein | *Fjoh_4501* | Function unknown | Periplasmic |  |  |  |  |  |
| Q8KRP0 | SPII | 39.28 | 2.31 | GldH | *Fjoh_0890* | Gliding/T9SS | OM lipoprotein |  |  |  |  |  |
| A5FHK8 | SPI | 20.38 | 2.26 | Peptidase family M1 possible nickel uptake system protein | *Fjoh_2281* | Inorganic ion transport and metabolism | Periplasmic |  |  |  |  |  |
| A5FEI9 | SPII | 22.23 | 2.25 | Predicted outer membrane protein | *Fjoh_3371* | Function unknown | OM lipoprotein |  |  |  |  |  |
| A5FM48 | SPI | 45.11 | 2.19 | Peptidase family S33 | *Fjoh_0689* | Lipid transport and metabolism | Periplasmic |  |  |  |  |  |
| A5FL26 | SPI | 41.91 | 2.06 | Peptidase subfamily M23B-like protein | *Fjoh_1067* | Cell cycle control, cell division, chromosome partitioning | Periplasmic |  |  |  |  |  |
| A5FK94 | SPII | 24.36 | 2.03 | Hypothetical lipoprotein | *Fjoh_1339* | Function unknown | OM lipoprotein |  |  |  |  |  |
| A5FHZ7 | SPI | 26.93 | 2 | Cytochrome c biogenesis protein, transmembrane region | *Fjoh_2139* | Energy production and conversion | Periplasmic |  |  |  |  |  |
| A5FJV6 | SPI | 24.7 | 1.96 | Uncharacterized protein | *Fjoh_1486* | Function unknown | Periplasmic |  |  |  |  |  |
| A5FJ14 | SPII | 24.36 | 1.94 | Hypothetical lipoprotein | *Fjoh_1777* | Function unknown | OM lipoprotein |  |  |  |  |  |
| A5FHJ6 | SPII | 35.17 | 1.94 | Hypothetical lipoprotein | *Fjoh_2300* | Function unknown | OM lipoprotein |  |  |  |  |  |
| A5FHQ3 | SPI | 35.82 | 1.93 | Tetratricopeptide TPR_2 repeat protein | *Fjoh_2246* | Function unknown | Periplasmic |  |  |  |  |  |
| A5FLC8 | SPI | 36.74 | 1.92 | PgPepO oligopeptidase peptidase family M13 | *Fjoh_0959* | Posttranslational modification, protein turnover, chaperones | Periplasmic |  |  |  |  |  |
| A5FJF0 | SPI | 22.64 | 1.89 | Sporulation domain protein | *Fjoh_1633* | Function unknown | Periplasmic |  |  |  |  |  |
| A5FMN9 | SPI | 23.48 | 1.84 | TPR repeat-containing protein | *Fjoh_0488* | Function unknown | Periplasmic |  |  |  |  |  |
| A5F9R8 | SPII | 20.61 | 1.83 | Hypothetical lipoprotein | *Fjoh_5055* | Function unknown | OM lipoprotein |  |  |  |  |  |
| A5FHZ0 | SPI | 23.1 | 1.8 | Putative esterase | *Fjoh_2146* | Function unknown | Periplasmic |  |  |  |  |  |
| A5FNV3 | SPII | 27.8 | 1.79 | Periplasmic binding protein | *Fjoh_0083* | Inorganic ion transport and metabolism | OM lipoprotein |  |  |  |  |  |
| A5FHC0 | SPII | 29.65 | 1.78 | Peptidylprolyl isomerase | *Fjoh_2368* | Posttranslational modification, protein turnover, chaperones | OM lipoprotein |  |  |  |  |  |
| A5FGY9 | SPII | 36.08 | 1.77 | Hypothetical lipoprotein | *Fjoh_2510* | Function unknown | OM lipoprotein |  |  |  |  |  |
| A5FKP3 | SPII-LES | 22.64 | 1.71 | Hypothetical lipoprotein | *Fjoh_1201* | Function unknown | Surface-exposed lipoprotein |  |  |  |  |  |
| A5F9Z8 | SPII | 28.45 | 1.67 | Lipocalin-like domain-containing protein | *Fjoh_4978* | Lipid transport and metabolism | OM lipoprotein |  |  |  |  |  |
| A5FMX9 | SPI | 30.21 | 1.65 | Uncharacterized protein | *Fjoh_0411* | Function unknown | Periplasmic |  |  |  |  |  |
| A5FIE5 | SPII-LES | 26.32 | 1.6 | Beta-xylanase | *Fjoh_1996* | Carbohydrate transport and metabolism | Surface-exposed lipoprotein | GH10 |  |  |  |  |
| A5FNC0 | SPII | 23.08 | 1.51 | DUF4369 domain-containing protein | *Fjoh_0264* | Function unknown | OM lipoprotein |  |  |  |  |  |
| A5FKB8 | SPI | 27.96 | 0.66 | SusC-like TonB-dependent receptor | *Fjoh_1314* | Carbohydrate transport and metabolism | OM integral protein | SusC |  |  |  |  |
| A5FAG3 | SPI | 20.7 | 0.65 | Uncharacterized conserved protein UCP016719 | *Fjoh_4806* | Function unknown | Periplasmic |  |  |  |  |  |
| A5FFP8 | SPI | 31.86 | 0.63 | Glutathione peroxidase | *Fjoh_2944* | Posttranslational modification, protein turnover, chaperones | Periplasmic |  |  |  |  |  |
| A5FGA4 | SPI | 23.14 | 0.62 | Adhesin domain-containing protein | *Fjoh_2750* | Cell wall/membrane/envelope biogenesis | Cell surface/extracellular |  |  |  |  |  |
| A5FE42 | SPI | 23.85 | 0.61 | Peptidase family M28 | *Fjoh_3518* | Function unknown | Periplasmic |  |  |  |  |  |
| A5FIR7 | SPII-LES | 29.73 | 0.6 | DNA-binding beta-propeller fold protein YncE | *Fjoh_1874* | Nucleotide transport and metabolism | Surface-exposed lipoprotein |  |  |  |  |  |
| A5FNF7 | SPI | 20.66 | 0.59 | Peptidase subfamily M48-like protein | *Fjoh_0225* | Cell wall/membrane/envelope biogenesis | Periplasmic |  |  |  |  |  |
| A5FLE9 | SPI | 39.17 | 0.59 | TonB-dependent receptor, plug | *Fjoh_0928* | Inorganic ion transport and metabolism | OM integral protein |  |  |  |  |  |
| A5FJZ8 | SPI | 24.06 | 0.59 | TonB-dependent receptor, plug | *Fjoh_1438* | Inorganic ion transport and metabolism | OM integral protein |  |  |  |  |  |
| A5FBC3 | SPI | 25.03 | 0.59 | SusC-like TonB-dependent receptor | *Fjoh_4500* | Carbohydrate transport and metabolism | OM integral protein | SusC | PUL34* |  |  |  |
| A5FG07 | SPII | 20.56 | 0.59 | Lipocalin-like domain-containing protein | *Fjoh_2832* | Lipid transport and metabolism | OM lipoprotein |  |  |  |  |  |
| A5FCM6 | SPI | 23.82 | 0.58 | AB hydrolase-1 domain-containing protein | *Fjoh_4027* | Function unknown | Periplasmic |  |  |  |  |  |
| A5FIZ8 | SPI | 44.7 | 0.58 | Outer membrane protein beta-barrel domain-containing protein | *Fjoh_1789* | Cell wall/membrane/envelope biogenesis | OM integral protein |  |  |  |  |  |
| A5FK08 | SPII | 22.79 | 0.58 | DUF4249 domain-containing protein | *Fjoh_1435* | Function unknown | OM lipoprotein |  |  |  |  |  |
| A5FBY8 | SPI | 21.34 | 0.57 | Isochorismatase hydrolase | *Fjoh_4273* | Secondary metabolites biosynthesis, transport and catabolism | Periplasmic |  |  |  |  |  |
| A5FB67 | SPI | 38.98 | 0.55 | SusC-like TonB-dependent receptor | *Fjoh_4559* | Carbohydrate transport and metabolism | OM integral protein | SusC | PUL30/34 & PUL35 |  |  |  |
| A5FHU7 | SPII | 33 | 0.55 | Cytochrome C | *Fjoh_2192* | Energy production and conversion | OM lipoprotein |  |  |  |  |  |
| A5FKE6 | SPI | 21.43 | 0.54 | Response regulator receiver protein | *Fjoh_1291* | Transcription | Periplasmic |  |  |  |  |  |
| A5FAE4 | SPII | 29.09 | 0.54 | Candidate beta-glycosidase Glycoside hydrolase family 3 | *Fjoh_4819* | Carbohydrate transport and metabolism | OM lipoprotein | Pept_SE/GH3 | PUL32 & PUL38 |  |  |  |
| A5FIT1 | SPI | 25.61 | 0.53 | GldN | *Fjoh_1856* | Gliding/T9SS | Periplasmic |  |  |  |  |  |
| A5FJJ6 | SPI | 22.99 | 0.53 | TonB-dependent receptor | *Fjoh_1588* | Inorganic ion transport and metabolism | OM integral protein |  |  |  |  |  |
| A5FJI1 | SPII-LES | 27.18 | 0.53 | Hypothetical lipoprotein | *Fjoh_1606* | Function unknown | Surface-exposed lipoprotein |  |  |  |  |  |
| A5FD24 | SPII-LES | 20.07 | 0.53 | RagB/SusD domain protein | *Fjoh_3881* | Carbohydrate transport and metabolism | Surface-exposed lipoprotein | SusD | PUL20 & PUL23 |  |  |  |
| A5FBC2 | SPII-LES | 24.38 | 0.53 | RagB/SusD domain protein | *Fjoh_4499* | Carbohydrate transport and metabolism | Surface-exposed lipoprotein | SusD | PUL34 |  |  |  |
| A5FFW4 | SPII-LES | 31.84 | 0.52 | DUF4302 domain-containing protein | *Fjoh_2891* | Function unknown | Surface-exposed lipoprotein |  |  |  |  |  |
| A5FFU9 | SPII-LES | 28.33 | 0.52 | RagB/SusD domain protein | *Fjoh_2893* | Carbohydrate transport and metabolism | Surface-exposed lipoprotein | SusD | PUL11 & PUL15 |  |  |  |
| A5FJY9 | SPI | 51.64 | 0.5 | Integral membrane sensor signal transduction histidine kinase | *Fjoh_1442* | Signal transduction mechanisms | IM lipoprotein |  |  |  |  |  |
| A5FEB7 | SPI | 24.91 | 0.5 | Outer membrane protein beta-barrel comain-containing protein | *Fjoh_3442* | Cell wall/membrane/envelope biogenesis | OM integral protein |  |  |  |  |  |
| A5FAF7 | SPI | 25.13 | 0.5 | Uncharacterized conserved protein UCP016719 | *Fjoh_4816* | Function unknown | Periplasmic |  |  |  |  |  |
| A5FCW3 | SPII-LES | 31.7 | 0.5 | RagB/SusD domain protein | *Fjoh_3944* | Carbohydrate transport and metabolism | Surface-exposed lipoprotein | SusD | PUL21 & PUL24 |  |  |  |
| A5FA28 | SPII | 21.25 | 0.5 | Hypothetical lipoprotein | *Fjoh_4940* | Function unknown | OM lipoprotein |  |  |  |  |  |
| A5FMI9 | SPI | 34.89 | 0.49 | Phosphate-selective porin O and P | *Fjoh_0542* | Inorganic ion transport and metabolism | OM integral protein |  |  |  |  |  |
| A5FK31 | SPI | 29.4 | 0.48 | SusC-like TonB-dependent receptor | *Fjoh_1405* | Carbohydrate transport and metabolism | OM integral protein | SusC | PUL3 & PUL6 |  |  |  |
| A5FA64 | SPI | 26.38 | 0.48 | Outer membrane efflux protein | *Fjoh_4902* | Cell wall/membrane/envelope biogenesis | OM integral protein |  |  |  |  |  |
| A5FJ27 | SPI | 50.04 | 0.48 | Uncharacterized protein | *Fjoh_1765* | Coenzyme transport and metabolism | OM integral protein |  |  |  |  |  |
| A5FM74 | SPII-LES | 30.32 | 0.48 | RagB/SusD domain protein | *Fjoh_0666* | Carbohydrate transport and metabolism | Surface-exposed lipoprotein | SusD | PUL3* |  |  |  |
| A5FFU8 | SPII | 24.76 | 0.48 | Substrate import-associated zinc metallohydrolase lipoprotein | *Fjoh_2892* | Function unknown | OM lipoprotein |  |  |  |  |  |
| A5FIS8 | SPII | 30.84 | 0.48 | GldK | *Fjoh_1853* | Gliding/T9SS | OM lipoprotein |  |  |  |  |  |
| A5FCU8 | SPI | 26.65 | 0.47 | Outer membrane protein-like protein | *Fjoh_3959* | Cell wall/membrane/envelope biogenesis | OM integral protein |  |  |  |  |  |
| A5FEH0 | SPI | 27.12 | 0.47 | Candidate beta-glycosidase Glycoside hydrolase family 3 | *Fjoh_3389* | Carbohydrate transport and metabolism | Periplasmic | GH3 |  |  |  |  |
| A5FJM5 | SPI | 32.5 | 0.47 | Candidate beta-glucosidase Glycoside hydrolase family 3 | *Fjoh_1567* | Carbohydrate transport and metabolism | Periplasmic | GH3 | PUL4 & PUL7 |  |  |  |
| A5FF76 | SPII-LES | 26.62 | 0.47 | RagB/SusD domain protein | *Fjoh_3126* | Carbohydrate transport and metabolism | Surface-exposed lipoprotein | SusD | PUL13 & PUL17 |  |  |  |
| A5FMK2 | SPI | 24.27 | 0.46 | Colicin import membrane protein | *Fjoh_0540* | Function unknown | Unknown |  |  | Yes |  |  |
| A5FLZ8 | SPI | 26.72 | 0.46 | SusC-like TonB-dependent receptor | *Fjoh_0736* | Carbohydrate transport and metabolism | OM integral protein | SusC | PUL3 & PUL6 |  |  |  |
| A5FKI3 | SPI | 30.74 | 0.45 | Alpha-ketoglutarate decarboxylase | *Fjoh_1257* | Energy production and conversion | OM integral protein |  |  |  |  |  |
| A5FGE4 | SPI | 22.76 | 0.45 | SusC-like TonB-dependent receptor | *Fjoh_2711* | Carbohydrate transport and metabolism | OM integral protein | SusC | PUL10 & PUL14 |  |  |  |
| A5FFV0 | SPI | 33.93 | 0.45 | SusC-like TonB-dependent receptor | *Fjoh_2894* | Carbohydrate transport and metabolism | OM integral protein | SusC | PUL11 & PUL15 |  |  |  |
| A5FB12 | SPI | 20.72 | 0.45 | Alpha/beta hydrolase fold protein | *Fjoh_4606* | Function unknown | Periplasmic |  |  |  |  |  |
| A5FAG6 | SPI | 27.03 | 0.45 | GHL10 domain-containing protein | *Fjoh_4809* | Function unknown | Periplasmic |  |  |  |  |  |
| A5FE81 | SPII | 35.85 | 0.45 | BamD | *Fjoh_3469* | Cell wall/membrane/envelope biogenesis | OM lipoprotein |  |  | Yes |  |  |
| A5FN99 | SPI | 32.45 | 0.44 | Peptidase family M24 | *Fjoh_0276* | Amino acid transport and metabolism | Periplasmic |  |  |  |  |  |
| A5FNF3 | SPI | 26 | 0.44 | BamB-like | *Fjoh_0237* | Cell wall/membrane/envelope biogenesis | Periplasmic |  |  |  |  |  |
| A5FA84 | SPII | 22.79 | 0.44 | Lipocalin-like domain-containing protein | *Fjoh_4891* | Lipid transport and metabolism | OM lipoprotein |  |  |  |  |  |
| A5FLQ6 | SPI | 27.65 | 0.43 | TonB-dependent receptor | *Fjoh_0821* | Inorganic ion transport and metabolism | OM integral protein |  |  |  |  |  |
| A5FKR4 | SPI | 35.63 | 0.43 | DUF3078 domain-containing protein | *Fjoh_1173* | Cell wall/membrane/envelope biogenesis | OM integral protein |  |  |  |  |  |
| A5FIL8 | SPI | 33.74 | 0.43 | SusC-like TonB-dependent receptor | *Fjoh_1924* | Carbohydrate transport and metabolism | OM integral protein | SusC | PUL8* |  |  |  |
| A5FJW3 | SPI | 24.6 | 0.42 | Carboxypeptidase-like regulatory domain-containing protein | *Fjoh_1476* | Cell wall/membrane/envelope biogenesis | OM integral protein |  |  |  |  |  |
| A5FJV5 | SPI | 33.28 | 0.42 | TonB-dependent receptor | *Fjoh_1485* | Inorganic ion transport and metabolism | OM integral protein |  |  |  |  |  |
| A5FH73 | SPI | 40.76 | 0.42 | FGE-sulfatase domain-containing protein | *Fjoh_2417* | Function unknown | Periplasmic |  |  |  |  |  |
| A5FFB9 | SPI | 30.29 | 0.42 | TonB-dependent receptor, plug | *Fjoh_3092* | Inorganic ion transport and metabolism | OM integral protein |  |  |  |  |  |
| A5FAG7 | SPI | 20.26 | 0.42 | Candidate esterase Carbohydrate esterase family 1 | *Fjoh_4810* | Function unknown | Periplasmic |  |  |  |  |  |
| A5FA54 | SPI | 34.14 | 0.42 | TonB-dependent receptor, plug | *Fjoh_4916* | Coenzyme transport and metabolism | OM integral protein |  |  |  |  |  |
| A5FAG5 | SPI | 21.68 | 0.42 | Beta-N-acetylhexosaminidase | *Fjoh_4808* | Carbohydrate transport and metabolism | Periplasmic | GH20 |  |  |  |  |
| A5FIN2 | SPII | 51.34 | 0.42 | DUF4136 domain-containing protein | *Fjoh_1907* | Function unknown | OM lipoprotein |  |  |  |  |  |
| A5FL78 | SPI | 28.46 | 0.41 | Uncharacterized protein | *Fjoh_1011* | Function unknown | Periplasmic |  |  |  |  |  |
| A5FJX0 | SPI | 29.85 | 0.41 | SprT | *Fjoh_1466* | Gliding/T9SS | OM integral protein |  |  |  |  |  |
| A5FH92 | SPI | 24.79 | 0.41 | Candidate d-4,5 unsaturated beta-glycuronidase Glycoside hydrolase family 88 | *Fjoh_2406* | Carbohydrate transport and metabolism | Periplasmic | GH88 |  |  |  |  |
| A5FGB5 | SPI | 29.5 | 0.41 | DUF4294 domain-containing protein | *Fjoh_2737* | Function unknown | Periplasmic |  |  |  |  |  |
| A5FE38 | SPI | 28.27 | 0.41 | Outermembrane protein | *Fjoh_3514* | Function unknown | OM integral protein |  |  |  |  |  |
| A5FB56 | SPI | 36.89 | 0.41 | SusC-like TonB-dependent receptor | *Fjoh_4562* | Carbohydrate transport and metabolism | OM integral protein | SusC | PUL30/34 & PUL35 |  |  |  |
| A5FK37 | SPI | 31.25 | 0.41 | Candidate alpha-glucosidase Glycoside hydrolase family 97 | *Fjoh_1400* | Carbohydrate transport and metabolism | Periplasmic | GH97 | PUL3 & CAZyme cluster 1 |  |  |  |
| A5FC59 | SPII-LES | 30.29 | 0.41 | RagB/SusD domain protein | *Fjoh_4195* | Carbohydrate transport and metabolism | Surface-exposed lipoprotein | SusD | PUL24 & PUL27 |  |  |  |
| A5FK70 | SPI | 25.11 | 0.4 | TonB-dependent receptor | *Fjoh_1368* | Inorganic ion transport and metabolism | OM integral protein |  |  |  |  |  |
| A5FIC9 | SPI | 31.5 | 0.4 | Peptidase family M16 domain protein | *Fjoh_2012* | Function unknown | Periplasmic |  |  |  |  |  |
| A5FFP7 | SPI | 34.19 | 0.4 | Hypothetical protein | *Fjoh_2958* | Cell wall/membrane/envelope biogenesis | OM integral protein |  |  |  |  |  |
| A5FHC7 | SPII | 27.8 | 0.4 | Peptidase family M61 domain protein | *Fjoh_2360* | Function unknown | OM lipoprotein |  |  |  |  |  |
| A5FA21 | SPII-LES | 35.63 | 0.4 | RagB/SusD domain protein | *Fjoh_4950* | Carbohydrate transport and metabolism | Surface-exposed lipoprotein | SusD | PUL33 & PUL39 |  |  |  |
| A5FBB1 | SPI | 28.06 | 0.39 | Glutathione hydrolase proenzyme | *Fjoh_4506* | Amino acid transport and metabolism | Periplasmic |  |  |  |  |  |
| A5FAK8 | SPI | 20.51 | 0.39 | OmpH family outer membrane protein | *Fjoh_4758* | Function unknown | Periplasmic |  |  |  |  |  |
| A5FAV4 | SPI | 28.9 | 0.39 | SusC-like TonB-dependent receptor | *Fjoh_4671* | Carbohydrate transport and metabolism | OM integral protein | SusC | PUL31 & PUL37 |  |  |  |
| A5FMB4 | SPI | 28.64 | 0.39 | GspD-like type II secretion system secretin protein | *Fjoh_0618* | Intracellular trafficking, secretion, and vesicular transport | Periplasmic |  |  |  |  |  |
| A5FJ92 | SPI | 52.11 | 0.38 | DUF6089 domain-containing protein | *Fjoh_1692* | Cell wall/membrane/envelope biogenesis | OM integral protein |  |  |  |  |  |
| A5FGD1 | SPII-LES | 21.91 | 0.38 | RagB/SusD domain protein | *Fjoh_2712* | Carbohydrate transport and metabolism | Surface-exposed lipoprotein | SusD | PUL10 & PUL14 |  |  |  |
| A5FLA7 | SPI | 28.54 | 0.37 | SprF | *Fjoh_0978* | Gliding/T9SS | OM integral protein |  |  |  |  |  |
| A5FF77 | SPI | 36.61 | 0.37 | SusC-like TonB-dependent receptor | *Fjoh_3127* | Carbohydrate transport and metabolism | OM integral protein | SusC | PUL13 & PUL17 |  |  |  |
| A5FDH2 | SPI | 27.26 | 0.37 | Alpha/beta hydrolase fold protein | *Fjoh_3736* | Lipid transport and metabolism | Periplasmic |  |  |  |  |  |
| A5FM73 | SPI | 31.93 | 0.36 | SusC-like TonB-dependent receptor | *Fjoh_0665* | Carbohydrate transport and metabolism | OM integral protein | SusC | PUL3 |  |  |  |
| A5FH56 | SPI | 28.11 | 0.36 | SusC-like TonB-dependent receptor | *Fjoh_2431* | Carbohydrate transport and metabolism | OM integral protein | SusC | PUL9 & PUL13 |  |  |  |
| A5FHX2 | SPII | 27.38 | 0.36 | Peptidase family S33 | *Fjoh_2171* | Lipid transport and metabolism | OM lipoprotein |  |  |  |  |  |
| A5F9W0 | SPI | 36.62 | 0.35 | Transporter | *Fjoh_5008* | Energy production and conversion | OM integral protein |  |  | Yes |  |  |
| A5FD66 | SPI | 63.62 | 0.35 | Carboxypeptidase regulatory-like domain-containing protein | *Fjoh_3841* | Inorganic ion transport and metabolism | OM integral protein |  |  |  |  |  |
| A5FK32 | SPII-LES | 27.43 | 0.35 | RagB/SusD domain protein | *Fjoh_1406* | Carbohydrate transport and metabolism | Surface-exposed lipoprotein | SusD | PUL3 & PUL6 |  |  |  |
| A5FJ03 | SPI | 50.9 | 0.34 | Porin | *Fjoh_1779* | Function unknown | OM integral protein |  |  |  |  |  |
| A5FBI5 | SPI | 47.7 | 0.34 | SusC-like TonB-dependent receptor | *Fjoh_4434* | Carbohydrate transport and metabolism | OM integral protein | SusC | PUL29 & PUL32 |  |  |  |
| A5FA22 | SPI | 33.27 | 0.34 | SusC-like TonB-dependent receptor | *Fjoh_4951* | Carbohydrate transport and metabolism | OM integral protein | SusC | PUL33 & PUL39 |  |  |  |
| A5FK33 | SPII | 48.26 | 0.34 | Hypothetical lipoprotein | *Fjoh_1407* | Function unknown | OM lipoprotein |  |  |  |  |  |
| A5FA20 | SPII-LES | 31.14 | 0.34 | IPT/TIG domain-containing protein | *Fjoh_4949* | Function unknown | Surface-exposed lipoprotein |  |  |  |  |  |
| A5FBS1 | SPI | 32.06 | 0.33 | DUF6377 domain-containing protein | *Fjoh_4332* | Transcription & Signal transduction mechanisms | Periplasmic |  |  |  |  |  |
| A5FA04 | SPI | 26.05 | 0.33 | NLP/P60 protein | *Fjoh_4966* | Cell wall/membrane/envelope biogenesis | Periplasmic |  |  |  |  |  |
| A5FC08 | SPI | 32.03 | 0.33 | SusC-like TonB-dependent receptor | *Fjoh_4255* | Carbohydrate transport and metabolism | OM integral protein | SusC | PUL26 & PUL29 |  |  |  |
| A5FFR0 | SPI | 29.63 | 0.33 | Outer membrane efflux protein | *Fjoh_2941* | Intracellular trafficking, secretion, and vesicular transport | OM integral protein |  |  |  |  |  |
| A5FMK1 | SPI | 54.79 | 0.32 | Uncharacterized protein | *Fjoh_0539* | Function unknown | OM integral protein |  |  |  |  |  |
| A5FNW0 | SPI | 34.21 | 0.32 | Endonuclease/exonuclease/phosphatase | *Fjoh_0074* | Function unknown | Periplasmic |  |  |  |  |  |
| A5FB55 | SPII-LES | 40.73 | 0.32 | RagB/SusD domain protein | *Fjoh_4561* | Carbohydrate transport and metabolism | Surface-exposed lipoprotein | SusD | PUL30/PUL34 & PUL35 |  |  |  |
| A5FAV5 | SPII-LES | 24.4 | 0.32 | RagB/SusD domain protein | *Fjoh_4672* | Carbohydrate transport and metabolism | Surface-exposed lipoprotein | SusD | PUL31 & PUL37 |  |  |  |
| A5FNK1 | SPI | 39.66 | 0.31 | SusC-like TonB-dependent receptor | *Fjoh_0185* | Carbohydrate transport and metabolism | OM integral protein | SusC | PUL1* |  |  |  |
| A5FKB7 | SPI | 21.35 | 0.31 | L, D-transpeptidase catalytic domain-containing protein | *Fjoh_1328* | Cell wall/membrane/envelope biogenesis | Periplasmic |  |  |  |  |  |
| A5FD40 | SPI | 26.48 | 0.29 | SusC-like TonB-dependent receptor | *Fjoh_3871* | Carbohydrate transport and metabolism | OM integral protein | SusC | PUL20 & PUL23 |  |  |  |
| A5FKG3 | SPI | 39.12 | 0.29 | PKD domain-containing protein | *Fjoh_1272* | Function unknown | OM integral protein |  |  |  |  |  |
| A5FM71 | SPII | 33.14 | 0.29 | Ig-like domain-containing protein | *Fjoh_0663* | Function unknown | OM lipoprotein |  |  |  |  |  |
| A5FE85 | SPI | 42.3 | 0.28 | Metallophosphoesterase | *Fjoh_3473* | Nucleotide transport and metabolism | Periplasmic |  |  |  |  |  |
| A5FEI0 | SPI | 41.36 | 0.27 | Outer membrane protein beta-barrel domain-containing protein | *Fjoh_3381* | Function unknown | OM integral protein |  |  |  |  |  |
| A5FH32 | SPI | 39.53 | 0.27 | TonB-dependent receptor | *Fjoh_2466* | Inorganic ion transport and metabolism | OM integral protein |  |  |  |  |  |
| A5FMB7 | SPII-LES | 31.79 | 0.27 | Fibronectin, type III domain protein | *Fjoh_0621* | Function unknown | Surface-exposed lipoprotein |  |  |  |  |  |
| A5FN23 | SPII | 52.97 | 0.27 | Wza | *Fjoh_0361* | Cell wall/membrane/envelope biogenesis | OM lipoprotein |  |  |  |  |  |
| A5FF33 | SPI | 27.23 | 0.26 | TonB-dependent receptor, plug | *Fjoh_3179* | Coenzyme transport and metabolism | OM integral protein |  |  |  |  |  |
| A5FC34 | SPI | 52.24 | 0.26 | TonB-dependent siderophore receptor | *Fjoh_4221* | Inorganic ion transport and metabolism | OM integral protein |  |  |  |  |  |
| A5FLI1 | SPII | 30.9 | 0.26 | Efflux transporter, RND family, MFP subunit | *Fjoh_0906* | Cell wall/membrane/envelope biogenesis | IM lipoprotein |  |  |  |  |  |
| A5FE84 | SPII | 41.66 | 0.26 | 5'-Nucleotidase domain protein | *Fjoh_3472* | Nucleotide transport and metabolism | OM lipoprotein |  |  |  |  |  |
| A5FGA3 | SPI | 50.97 | 0.25 | Putative auto-transporter adhesin head GIN domain-containing protein | *Fjoh_2749* | Cell wall/membrane/envelope biogenesis | Cell surface/extracellular |  |  |  |  |  |
| A5FJM0 | SPI | 30.16 | 0.24 | Candidate beta-glycosidase Glycoside hydrolase family 30 | *Fjoh_1562* | Carbohydrate transport and metabolism | Periplasmic | GH30_1 | PUL4 & PUL7 |  |  |  |
| A5FCM0 | SPI | 38.29 | 0.24 | TonB-dependent siderophore receptor | *Fjoh_4039* | Inorganic ion transport and metabolism | OM integral protein |  |  |  |  |  |
| A5FJM2 | SPI | 41.81 | 0.24 | Candidate beta-glycosidase Glycoside hydrolase family 3 | *Fjoh_1564* | Carbohydrate transport and metabolism | Periplasmic | GH3 | PUL4 & PUL7 |  |  |  |
| A5FNK0 | SPII-LES | 45.09 | 0.24 | RagB/SusD domain protein | *Fjoh_0184* | Carbohydrate transport and metabolism | Surface-exposed lipoprotein |  | PUL1 |  |  |  |
| A5FAV9 | SPII | 51.84 | 0.24 | Peptidase family M28 | *Fjoh_4661* | Function unknown | OM lipoprotein |  |  |  |  |  |
| A5FEN2 | SPI | 21.62 | 0.23 | TonB-dependent siderophore receptor | *Fjoh_3320* | Inorganic ion transport and metabolism | OM integral protein |  |  |  |  |  |
| A5FNJ9 | SPII-LES | 51.1 | 0.23 | Fibronectin, type III domain protein | *Fjoh_0183* | Function unknown | Surface-exposed lipoprotein |  |  |  |  |  |
| A5FNC9 | SPII | 62.84 | 0.23 | Lipocalin-like domain-containing protein | *Fjoh_0259* | Lipid transport and metabolism | OM lipoprotein |  |  |  |  |  |
| A5FND5 | SPI | 30.58 | 0.22 | TonB-dependent receptor | *Fjoh_0249* | Inorganic ion transport and metabolism | OM integral protein |  |  |  |  |  |
| A5FEE5 | SPI | 33.44 | 0.22 | Outer membrane protein beta-barrel domain-containing protein | *Fjoh_3401* | Function unknown | OM integral protein |  |  |  |  |  |
| A5FC74 | SPI | 49.35 | 0.22 | SusC-like TonB-dependent receptor | *Fjoh_4194* | Carbohydrate transport and metabolism | OM integral protein | SusC | PUL24 & PUL27 |  |  |  |
| A5FJN2 | SPI | 55.26 | 0.22 | SusC-like TonB-dependent receptor | *Fjoh_1560* | Carbohydrate transport and metabolism | OM integral protein | SusC | PUL4 & PUL7 |  |  |  |
| A5FKQ1 | SPII | 64.18 | 0.22 | Hypothetical lipoprotein | *Fjoh_1177* | Posttranslational modification, protein turnover, chaperones | OM lipoprotein |  |  |  |  |  |
| A5FJM4 | SPII | 38.55 | 0.22 | Candidate beta-glycosidase Glycoside hydrolase family 30 | *Fjoh_1566* | Carbohydrate transport and metabolism | OM lipoprotein | GH30_3 | PUL4 & PUL7 |  |  |  |
| A5FB06 | SPII | 41.3 | 0.21 | META domain-containing protein | *Fjoh_4623* | Posttranslational modification, protein turnover, chaperones | OM lipoprotein |  |  |  |  |  |
| A5FJM1 | SPII | 34.41 | 0.2 | Candidate beta-glycosidase Glycoside hydrolase family 30 | *Fjoh_1563* | Carbohydrate transport and metabolism | Periplasmic | GH30_1 | PUL4 & PUL7 |  |  |  |
| A5FGL7 | SPII | 27.25 | 0.2 | 3-keto-disaccharide hydrolase domain-containing protein | *Fjoh_2621* | Function unknown | OM lipoprotein |  |  |  |  |  |
| A5FE35 | SPII-LES | 38.24 | 0.19 | RagB/SusD domain protein | *Fjoh_3524* | Carbohydrate transport and metabolism | Surface-exposed lipoprotein | SusD | PUL16 & PUL20 |  |  |  |
| A5FIM2 | SPII | 80.22 | 0.18 | Peptidoglycan hydrolase | *Fjoh_1913* | Cell wall/membrane/envelope biogenesis | OM lipoprotein |  |  |  |  |  |
| A5FJ15 | SPI | 25.49 | 0.17 | Lipid/polyisoprenoid-binding YceI-like domain-containing protein | *Fjoh_1778* | Function unknown | Periplasmic |  |  |  |  |  |
| A5FEK0 | SPI | 42.73 | 0.17 | Outer membrane protein beta-barrel domain-containing protein | *Fjoh_3350* | Cell wall/membrane/envelope biogenesis | OM integral protein |  |  |  |  |  |
| A5FEL3 | SPI | 34.89 | 0.17 | Uncharacterized protein | *Fjoh_3349* | Function unknown | OM integral protein |  |  |  |  |  |
| A5FCV6 | SPI | 49.23 | 0.16 | SprF-like protein | *Fjoh_3951* | Gliding/T9SS | OM integral protein |  |  |  |  |  |
| A5FNS6 | SPI | 52.43 | 0.15 | Phosphate-selective porin O and P | *Fjoh_0105* | Function unknown | OM integral protein |  |  | Yes |  |  |
| A5FE72 | SPI | 35.59 | 0.15 | SprF-like protein | *Fjoh_3477* | Gliding/T9SS | OM integral protein |  |  |  |  |  |
| A5FE36 | SPI | 53.19 | 0.13 | SusC-like TonB-dependent receptor | *Fjoh_3525* | Carbohydrate transport and metabolism | OM integral protein | SusC | PUL16 & PUL20 |  |  |  |
| A5FMS1 | SPII | 47.68 | 0.13 | Peptidase family M28 | *Fjoh_0454* | Function unknown | OM lipoprotein |  |  |  |  |  |
| A5FJZ6 | SPII | 55.44 | 0.13 | Hypothetical lipoprotein | *Fjoh_1436* | Function unknown | OM lipoprotein |  |  |  |  |  |
| A5FBD6 | SPI | 43.97 | 0.1 | Outer membrane efflux protein | *Fjoh_4485* | Intracellular trafficking, secretion, and vesicular transport | OM integral protein |  |  |  |  |  |
| A5FI22 | SPI | 76.55 | 0.1 | Possible lipoprotein carrier protein LolA | *Fjoh_2111* | Cell wall/membrane/envelope biogenesis | Periplasmic |  |  |  |  |  |
| A5FNC8 | SPII | 62.58 | 0.1 | OmpA/MotB domain protein | *Fjoh_0258* | Cell wall/membrane/envelope biogenesis | OM lipoprotein |  |  |  |  |  |
| A5FAL0 | SPII | 60.41 | 0.1 | META domain-containing protein | *Fjoh_4760* | Posttranslational modification, protein turnover, chaperones | OM lipoprotein |  |  |  |  |  |
| A5FHI0 | SPI | 54.95 | 0.07 | OmpA/MotB domain protein | *Fjoh_2321* | Cell wall/membrane/envelope biogenesis | OM integral protein |  |  |  |  |  |
| A1E5U4 | SPI | 28.23 | 0.05 | SprD | *Fjoh_0980* | Gliding/T9SS | Unknown |  |  |  |  |  |
| A5FJM9 | SPII | 51.16 | 0.05 | GldJ | *Fjoh_1557* | Gliding/T9SS | OM lipoprotein |  |  |  |  |  |
| A5FFC2 | SPI | 47.4 | 0.02 | DUF2911 domain-containing protein | *Fjoh_3076* | Function unknown | Periplasmic |  |  |  |  |  |
| A5FK70 | SPI | 52.17 | 0 | TonB-dependent receptor | *Fjoh_1368* | Inorganic ion transport and metabolism | OM integral protein |  |  |  |  |  |
| A5FG24 | SPI | 135.31 | 0 | Choloylglycine hydrolase peptidase family C59 | *Fjoh_2831* | Cell wall/membrane/envelope biogenesis | Periplasmic |  |  |  |  |  |
| A5FBC7 | SPII-LES | 117.53 | 0 | RagB/SusD domain protein | *Fjoh_4490* | Polysaccharide utilization | Surface-exposed lipoprotein | SusD | PUL33 |  |  |  |
| Δ*lolB1* | | | | | | | | | | | |  |
| Accession | **Signal peptide** | **Significance** | ***lolB1*/WT FC** | **Description** | **Gene code** | **COG Class Description** | **Localization** | **CAZy family** | **PUL (literature and CAZy prediction)** | **Essentiality** |  |  |
| A5FC16 | SPII-LES | 115.07 | 64 | Peptidase family M57 | *Fjoh_4241* | Posttranslational modification, protein turnover, chaperones | OM lipoprotein |  |  |  |  |  |
| A5FHF7 | SPII | 54.84 | 48.29 | Hypothetical lipoprotein | *Fjoh_2342* | Function unknown | OM lipoprotein |  |  |  |  |  |
| A5FNT4 | SPI | 70.41 | 29.07 | DUF4252 domain-containing protein | *Fjoh_0096* | Function unknown | Periplasmic |  |  |  |  |  |
| A5FNT5 | SPII | 49.59 | 18.55 | Hypothetical lipoprotein | *Fjoh_0097* | Function unknown | OM lipoprotein |  |  |  |  |  |
| A5FDE8 | SPI | 46.23 | 8.22 | DUF3570 domain-containing protein | *Fjoh_3759* | Lipid transport and metabolism | OM integral protein |  |  |  |  |  |
| A5FF13 | SPII-LES | 35.45 | 6.36 | Hypothetical lipoprotein | *Fjoh_3186* | Function unknown | Surface-exposed lipoprotein |  |  |  |  |  |
| A5FAC1 | SPII-LES | 48.44 | 5.83 | Lipocalin-like domain-containing protein | *Fjoh_4843* | Lipid transport and metabolism | Surface-exposed lipoprotein |  |  |  |  |  |
| A5FHZ5 | SPII | 72.66 | 5.67 | Hypothetical lipoprotein | *Fjoh_2151* | Function unknown | OM lipoprotein |  |  |  |  |  |
| A5FNR2 | SPI | 55.74 | 5.27 | OMP_b-brl_2 domain-containing protein | *Fjoh_0127* | Cell wall/membrane/envelope biogenesis | OM integral protein |  |  |  |  |  |
| A5F9V9 | SPII-LES | 47.43 | 3.53 | Cytochrome-c peroxidase | *Fjoh_5007* | Energy production and conversion | Surface-exposed lipoprotein |  |  |  |  |  |
| A5F9R8 | SPII | 38.34 | 3.2 | Hypothetical lipoprotein | *Fjoh_5055* | Function unknown | OM lipoprotein |  |  |  |  |  |
| A5FHA6 | SPI | 43.83 | 3.17 | Antitoxin component YwqK of YwqJK toxin-antitoxin module | *Fjoh_2385* | Function unknown | Periplasmic |  |  |  |  |  |
| A5FNB6 | SPII | 27.56 | 3.02 | META domain-containing protein | *Fjoh_0275* | Posttranslational modification, protein turnover, chaperones | OM lipoprotein |  |  |  |  |  |
| A5FND7 | SPI | 36.13 | 2.9 | TonB-dependent receptor, plug | *Fjoh_0252* | Inorganic ion transport and metabolism | OM integral protein |  |  |  |  |  |
| A5FAK9 | SPI | 35.49 | 2.9 | DUF4251 domain-containing protein | *Fjoh_4759* | Function unknown | Periplasmic |  |  |  |  |  |
| A5FB40 | SPI | 34.29 | 2.74 | Outer membrane protein beta-barrel domain-containing protein | *Fjoh_4578* | Cell wall/membrane/envelope biogenesis | OM integral protein |  |  |  |  |  |
| A5FJ27 | SPI | 44.08 | 2.47 | Uncharacterized protein | *Fjoh_1765* | Coenzyme transport and metabolism | OM integral protein |  |  |  |  |  |
| A5FAG3 | SPI | 32.39 | 2.36 | Uncharacterized conserved protein UCP016719 | *Fjoh_4806* | Function unknown | Periplasmic |  |  |  |  |  |
| A5FNX9 | SPII | 31.59 | 2.26 | Peptidylprolyl isomerase | *Fjoh_0050* | Posttranslational modification, protein turnover, chaperones | OM lipoprotein |  |  |  |  |  |
| A5FLD6 | SPII | 26.42 | 2.2 | DUF4421 domain-containing protein | *Fjoh_0952* | Cell motility | OM lipoprotein | SusD | PUL26 & PUL29 |  |  |  |
| A5FC07 | SPII-LES | 24.35 | 2.2 | RagB/SusD domain protein | *Fjoh_4254* | Carbohydrate transport and metabolism | Surface-exposed lipoprotein | SusC | PUL26 & PUL29 |  |  |  |
| A5FC08 | SPI | 23.28 | 2.16 | SusC-like TonB-dependent receptor | *Fjoh_4255* | Carbohydrate transport and metabolism | OM integral protein |  |  |  |  |  |
| A5FAF7 | SPI | 32.54 | 2.15 | Uncharacterized conserved protein UCP016719 | *Fjoh_4816* | Function unknown | Periplasmic |  |  |  |  |  |
| A5FC53 | SPII | 27.88 | 2.13 | Copper homeostasis protein | *Fjoh_4206* | Cell wall/membrane/envelope biogenesis | OM lipoprotein |  |  |  |  |  |
| A5FEP9 | SPII-LES | 23.07 | 2.13 | Glucose sorbosone dehydrogenase | *Fjoh_3307* | Carbohydrate transport and metabolism | Surface-exposed lipoprotein |  |  |  |  |  |
| A5FAG0 | SPII-LES | 22.68 | 2.09 | Hypothetical lipoprotein | *Fjoh_4803* | Function unknown | Surface-exposed lipoprotein | SusD | PUL22 & PUL25 |  |  |  |
| A5FCG9 | SPII-LES | 36.91 | 2.08 | RagB/SusD domain protein | *Fjoh_4094* | Carbohydrate transport and metabolism | Surface-exposed lipoprotein |  |  |  |  |  |
| A5FMH4 | SPII-LES | 22.25 | 2.06 | Hypothetical lipoprotein | *Fjoh_0561* | Function unknown | Surface-exposed lipoprotein |  |  |  |  |  |
| A5FNS8 | SPI | 34.61 | 2.05 | PepSY_like domain-containing protein | *Fjoh_0107* | Function unknown | Periplasmic |  |  |  |  |  |
| A5FI22 | SPI | 70.99 | 2.04 | Possible lipoprotein carrier protein LolA | *Fjoh_2111* | Cell wall/membrane/envelope biogenesis | Periplasmic |  |  |  |  |  |
| A5FLY5 | SPII-LES | 21.6 | 2.04 | PKD domain containing protein | *Fjoh_0757* | Function unknown | Surface-exposed lipoprotein |  |  |  |  |  |
| A5FAI4 | SPII | 32.55 | 2.03 | DUF3300 domain-containing protein | *Fjoh_4779* | RNA processing and modification | OM lipoprotein |  |  |  |  |  |
| A5FMN5 | SPI | 30.98 | 1.99 | Beta-lactamase peptidase family S12 | *Fjoh_0500* | Defense mechanisms | Periplasmic |  |  |  |  |  |
| A5FGA3 | SPI | 55.98 | 1.98 | Putative auto-transporter adhesin head GIN domain-containing protein | *Fjoh_2749* | Cell wall/membrane/envelope biogenesis | Cell surface/extracellular |  |  |  |  |  |
| A5FEZ9 | SPI | 20.4 | 1.93 | Uncharacterized protein | *Fjoh_3206* | Function unknown | Periplasmic |  |  |  |  |  |
| A5FMK7 | SPII | 20.62 | 1.91 | Hypothetical lipoprotein | *Fjoh_0527* | Function unknown | OM lipoprotein | GH105 | PUL26 & PUL29 |  |  |  |
| A5FC14 | SPII | 27.09 | 1.9 | Candidate d-4,5-unsaturated beta-glycuronidase Glycoside hydrolase family 105 | *Fjoh_4250* | Carbohydrate transport and metabolism | OM lipoprotein | SusC | PUL6 & PUL10 |  |  |  |
| A5FIC3 | SPI | 29.73 | 1.89 | SusC-like TonB-dependent receptor | *Fjoh_2020* | Carbohydrate transport and metabolism | OM integral protein | SusC | PUL20 & PUL23 |  |  |  |
| A5FD25 | SPI | 23.16 | 1.86 | SusC-like TonB-dependent receptor | *Fjoh_3882* | Carbohydrate transport and metabolism | OM integral protein |  |  |  |  |  |
| A5FHB6 | SPI | 40.82 | 1.86 | Peptidoglycan-binding LysM | *Fjoh_2379* | Cell wall/membrane/envelope biogenesis | Periplasmic |  |  |  |  |  |
| A5FJB8 | SPI | 27.43 | 1.85 | SprF-like protein | *Fjoh_1677* | Gliding/T9SS | OM integral protein |  |  |  |  |  |
| A5FHQ3 | SPI | 29.74 | 1.85 | Tetratricopeptide TPR_2 repeat protein | *Fjoh_2246* | Function unknown | Periplasmic |  |  |  |  |  |
| A5FK94 | SPII-LES | 22 | 1.81 | Hypothetical lipoprotein | *Fjoh_1339* | Function unknown | Surface-exposed lipoprotein |  |  |  |  |  |
| A5FJV3 | SPI | 24.15 | 1.8 | Glutamine cyclotransferase | *Fjoh_1483* | Function unknown | Periplasmic |  |  |  |  |  |
| A5FGG2 | SPII | 23.3 | 1.8 | Hypothetical lipoprotein | *Fjoh_2679* | Function unknown | OM lipoprotein |  |  |  |  |  |
| A5FL98 | SPI | 21.32 | 1.78 | Uncharacterized protein | *Fjoh_0984* | Function unknown | Periplasmic |  |  |  |  |  |
| A5FEL3 | SPI | 30.21 | 1.78 | Uncharacterized protein | *Fjoh_3349* | Function unknown | OM integral protein |  |  |  |  |  |
| A5FMN9 | SPI | 30.21 | 1.77 | TPR repeat-containing protein | *Fjoh_0488* | Function unknown | Periplasmic |  |  |  |  |  |
| A5FLC8 | SPI | 37.81 | 1.76 | PgPepO oligopeptidase peptidase family M13 | *Fjoh_0959* | Posttranslational modification, protein turnover, chaperones | Periplasmic |  |  |  |  |  |
| A5FFP7 | SPI | 23.3 | 1.76 | Hypothetical protein | *Fjoh_2958* | Cell wall/membrane/envelope biogenesis | OM integral protein |  |  |  |  |  |
| A5FD35 | SPII | 22.37 | 1.76 | Lipolytic enzyme, G-D-S-L family | *Fjoh_3879* | Amino acid transport and metabolism | OM lipoprotein |  |  |  |  |  |
| A5FF07 | SPI | 23.81 | 1.66 | Gliding motility protein RemI | *Fjoh_3194* | Gliding/T9SS | Cell surface/extracellular |  |  |  |  |  |
| A5FKG3 | SPI | 25.81 | 1.66 | PKD domain-containing protein | *Fjoh_1272* | Function unknown | OM integral protein |  |  |  |  |  |
| A5FH76 | SPII | 20.43 | 1.66 | Hypothetical lipoprotein | *Fjoh_2420* | Function unknown | OM lipoprotein |  |  |  |  |  |
| A5FNT1 | SPII-LES | 37.49 | 1.65 | Peptidase S41, subfamily S41A unassigned peptidases | *Fjoh_0093* | Cell wall/membrane/envelope biogenesis | Surface-exposed lipoprotein | Pept_SE/GH3 | PUL32 & PUL38 |  |  |  |
| A5FAE4 | SPII | 22.82 | 1.64 | Candidate beta-glycosidase Glycoside hydrolase family 3 | *Fjoh_4819* | Carbohydrate transport and metabolism | OM lipoprotein |  |  |  |  |  |
| A5FH32 | SPI | 24.45 | 1.59 | TonB-dependent receptor | *Fjoh_2466* | Inorganic ion transport and metabolism | OM integral protein |  |  |  |  |  |
| A5FMW8 | SPI | 20.97 | 1.57 | Dipeptidyl-peptidase | *Fjoh_0416* | Amino acid transport and metabolism | Periplasmic |  |  |  |  |  |
| A5FJV4 | SPI | 21.37 | 1.55 | GLPGLI family protein | *Fjoh_1484* | Function unknown | Unknown |  |  |  |  |  |
| A5FF84 | SPI | 28.19 | 1.54 | DUF4861 domain-containing protein | *Fjoh_3122* | Function unknown | Periplasmic |  |  |  |  |  |
| A5FC67 | SPI | 29.12 | 1.52 | DUF4861 domain-containing protein | *Fjoh_4187* | Function unknown | Periplasmic |  |  |  |  |  |
| A5FJM8 | SPI | 33.5 | 1.51 | Por secretion system protein PorU precursor. C-terminal signal peptidase | *Fjoh_1556* | Gliding/T9SS | OM protein |  |  |  |  |  |
| A5FGM2 | SPI | 22.13 | 1.51 | Peptidase family S33-like protein | *Fjoh_2626* | Lipid transport and metabolism | Periplasmic | GH20 |  |  |  |  |
| A5FAG5 | SPI | 21.13 | 1.5 | Beta-N-acetylhexosaminidase | *Fjoh_4808* | Carbohydrate transport and metabolism | Periplasmic |  |  |  |  |  |
| A5FIZ8 | SPI | 21.74 | 0.66 | Outer membrane protein beta-barrel domain-containing protein | *Fjoh_1789* | Cell wall/membrane/envelope biogenesis | OM integral protein |  |  |  |  |  |
| A5FIR6 | SPII-LES | 31.11 | 0.66 | PKD domain containing protein | *Fjoh_1873* | Function unknown | Surface-exposed lipoprotein |  |  |  |  |  |
| A5FCU8 | SPI | 28.83 | 0.64 | Outer membrane protein-like protein | *Fjoh_3959* | Cell wall/membrane/envelope biogenesis | OM integral protein |  |  |  |  |  |
| A5FJ10 | SPI | 24.1 | 0.63 | Aminopeptidase N-like protein | *Fjoh_1773* | Amino acid transport and metabolism | Periplasmic |  |  |  |  |  |
| A5FHK9 | SPI | 35.82 | 0.62 | TonB-dependent siderophore receptor | *Fjoh_2282* | Inorganic ion transport and metabolism | OM integral protein |  |  |  |  |  |
| A5FJJ6 | SPI | 23.09 | 0.6 | TonB-dependent receptor | *Fjoh_1588* | Inorganic ion transport and metabolism | OM integral protein |  |  |  |  |  |
| A5FCM6 | SPI | 21.43 | 0.59 | AB hydrolase-1 domain-containing protein | *Fjoh_4027* | Function unknown | Periplasmic |  |  |  |  |  |
| A5FBD6 | SPI | 22.02 | 0.58 | Outer membrane efflux protein | *Fjoh_4485* | Intracellular trafficking, secretion, and vesicular transport | OM integral protein |  |  |  |  |  |
| A5FNF3 | SPI | 26.25 | 0.57 | BamB-like | *Fjoh_0237* | Cell wall/membrane/envelope biogenesis | Periplasmic |  |  |  |  |  |
| A5FL26 | SPI | 36.94 | 0.55 | Peptidase subfamily M23B-like protein | *Fjoh_1067* | Cell cycle control, cell division, chromosome partitioning | Periplasmic |  |  |  |  |  |
| A5FD66 | SPI | 24.61 | 0.55 | Carboxypeptidase regulatory-like domain-containing protein | *Fjoh_3841* | Inorganic ion transport and metabolism | OM integral protein | GH3 | PUL4 & PUL7 |  |  |  |
| A5FJM5 | SPI | 37.69 | 0.53 | Candidate beta-glucosidase Glycoside hydrolase family 3 | *Fjoh_1567* | Carbohydrate transport and metabolism | Periplasmic | SusD | PUL34 |  |  |  |
| A5FBC2 | SPII-LES | 25.79 | 0.51 | RagB/SusD domain protein | *Fjoh_4499* | Carbohydrate transport and metabolism | Surface-exposed lipoprotein | GH97 | PUL3 & CAZyme cluster 1 |  |  |  |
| A5FK37 | SPI | 34.98 | 0.48 | Candidate alpha-glucosidase Glycoside hydrolase family 97 | *Fjoh_1400* | Carbohydrate transport and metabolism | Periplasmic |  |  |  |  |  |
| A5FN31 | SPI | 24.15 | 0.44 | Polysaccharide export protein | *Fjoh_0353* | Cell wall/membrane/envelope biogenesis | Periplasmic | SusC | PUL31 & PUL37 |  |  |  |
| A5FAV4 | SPI | 30.31 | 0.44 | SusC-like TonB-dependent receptor | *Fjoh_4671* | Carbohydrate transport and metabolism | OM integral protein | SusD | PUL10 & PUL14 |  |  |  |
| A5FGD1 | SPII-LES | 26.22 | 0.41 | RagB/SusD domain protein | *Fjoh_2712* | Carbohydrate transport and metabolism | Surface-exposed lipoprotein |  |  |  |  |  |
| A5FNV5 | SPII-LES | 36.62 | 0.39 | PrcB C-terminal domain-containing protein | *Fjoh_0069* | Function unknown | Surface-exposed lipoprotein | GH3 |  |  |  |  |
| A5FEH0 | SPI | 34.7 | 0.34 | Candidate beta-glycosidase Glycoside hydrolase family 3 | *Fjoh_3389* | Carbohydrate transport and metabolism | Periplasmic |  |  |  |  |  |
| A5FIT1 | SPI | 52.46 | 0.33 | GldN | *Fjoh_1856* | Gliding/T9SS | Periplasmic |  |  |  |  |  |
| A1E5U5 | SPI | 47.7 | 0.32 | SprB | *Fjoh_0979* | Gliding/T9SS | Surface-exposed |  |  |  |  |  |
| A5FHG9 | SPII | 45.05 | 0.32 | DUF4197 domain-containing protein | *Fjoh_2327* | Function unknown | OM lipoprotein |  |  |  |  |  |
| A5FJZ6 | SPII | 54.55 | 0.31 | Hypothetical lipoprotein | *Fjoh_1436* | Function unknown | OM lipoprotein |  |  |  |  |  |
| A5FMB7 | SPII-LES | 45.03 | 0.29 | Fibronectin, type III domain protein | *Fjoh_0621* | Function unknown | Surface-exposed lipoprotein |  |  |  |  |  |
| A5FMC4 | SPI | 64.4 | 0.28 | Uncharacterized protein | *Fjoh_0610* | Function unknown | Periplasmic |  |  |  |  |  |
| A5FIM2 | SPII | 58.81 | 0.28 | Peptidoglycan hydrolase | *Fjoh_1913* | Cell wall/membrane/envelope biogenesis | OM lipoprotein |  |  |  |  |  |
| A5FJZ7 | SPII | 32.54 | 0.27 | DUF4249 domain-containing protein | *Fjoh_1437* | Function unknown | OM lipoprotein | GH30_1 | PUL4 & PUL7 |  |  |  |
| A5FJM1 | SPII | 32.72 | 0.26 | Candidate beta-glycosidase Glycoside hydrolase family 30 | *Fjoh_1563* | Carbohydrate transport and metabolism | Periplasmic |  |  |  |  |  |
| A5FMB4 | SPI | 57.53 | 0.24 | GspD-like type II secretion system secretin protein | *Fjoh_0618* | Intracellular trafficking, secretion, and vesicular transport | Periplasmic |  |  |  |  |  |
| A5FFR0 | SPI | 50.84 | 0.24 | Outer membrane efflux protein | *Fjoh_2941* | Intracellular trafficking, secretion, and vesicular transport | OM integral protein |  |  |  |  |  |
| A5FN23 | SPII | 62.52 | 0.23 | Wza | *Fjoh_0361* | Cell wall/membrane/envelope biogenesis | OM lipoprotein |  |  |  |  |  |
| A5FJM9 | SPII | 51.32 | 0.22 | GldJ | *Fjoh_1557* | Gliding/T9SS | OM lipoprotein | SusD | PUL31 & PUL37 |  |  |  |
| A5FAV5 | SPII-LES | 42.6 | 0.21 | RagB/SusD domain protein | *Fjoh_4672* | Carbohydrate transport and metabolism | Surface-exposed lipoprotein | SusC | PUL4 & PUL7 |  |  |  |
| A5FJN2 | SPI | 64.36 | 0.2 | SusC-like TonB-dependent receptor | *Fjoh_1560* | Carbohydrate transport and metabolism | OM integral protein |  |  |  |  |  |
| A5FHI9 | SPI | 40.57 | 0.19 | DUF5723 domain-containing protein | *Fjoh_2310* | Cell wall/membrane/envelope biogenesis | OM integral protein | SusD | PUL4 & PUL7 |  |  |  |
| A5FJL9 | SPII-LES | 66.23 | 0.18 | RagB/SusD domain protein | *Fjoh_1561* | Carbohydrate transport and metabolism | Surface-exposed lipoprotein |  |  |  |  |  |
| A5FJ38 | SPI | 44.7 | 0.17 | Outer membrane protein beta-barrel domain-containing protein | *Fjoh_1745* | Cell wall/membrane/envelope biogenesis | OM integral protein |  |  |  |  |  |
| A5FCV6 | SPI | 47.24 | 0.16 | SprF-like protein | *Fjoh_3951* | Gliding/T9SS | OM integral protein |  |  |  |  |  |
| A5FNW0 | SPI | 61.25 | 0.15 | Endonuclease/exonuclease/phosphatase | *Fjoh_0074* | Function unknown | Periplasmic | GH3 | PUL4 & PUL7 |  |  |  |
| A5FJM2 | SPI | 47.09 | 0.13 | Candidate beta-glycosidase Glycoside hydrolase family 3 | *Fjoh_1564* | Carbohydrate transport and metabolism | Periplasmic |  |  |  |  |  |
| A5FHI0 | SPI | 58.23 | 0.08 | OmpA/MotB domain protein | *Fjoh_2321* | Cell wall/membrane/envelope biogenesis | OM integral protein |  |  |  |  |  |
| A5FIS8 | SPII | 59.6 | 0.08 | GldK | *Fjoh_1853* | Gliding/T9SS | OM lipoprotein |  |  |  |  |  |
| A5FL25 | SPII | 81.18 | 0.02 | Hypothetical lipoprotein | *Fjoh_1066* | Function unknown | OM lipoprotein |  |  |  |  |  |
