# Supplementary material for: LolA and LolB are conserved in Bacteroidota and are crucial for gliding motility and Type IX secretion
Source: Commun Biol. 2025 Mar 6;8:376. doi: 10.1038/s42003-025-07817-2 (PMC11885536; doi:10.1038/s42003-025-07817-2)
Supplement: Supplementary file 5 — Supplementary Data 2 [file 42003_2025_7817_MOESM5_ESM.docx]

**Supplementary Data 2. Cytoplasmic and IM proteins detected in the OM of the *lolA1* and *lolB1* mutants (FC ≥ 1.5, significance ≥ 20).**

**Δ*lolA1***

| Accession | Significance | Δ*lolA1*/WT FC | Description | Gene code |
| --- | --- | --- | --- | --- |
| A5FNJ4 | 71.29 | 26.09 | Uncharacterized protein | *Fjoh_0194* |
| A5FCD3 | 20.28 | 11.73 | Transporter, hydrophobe/amphiphile efflux-1 (HAE1) family | *Fjoh_4131* |
| A5FI28 | 54.07 | 11.27 | Alkyl hydroperoxide reductase/ Thiol specific antioxidant/ Mal allergen | *Fjoh_2117* |
| A5FNI3 | 58.02 | 8.72 | Phage shock protein C, PspC | *Fjoh_0196* |
| A5FH88 | 25.79 | 7.77 | DEAD/DEAH box helicase domain protein | *Fjoh_2402* |
| A5FEC7 | 44.06 | 7.68 | Argininuccinate synthase | *Fjoh_3435* |
| A5FLJ0 | 68.37 | 6.88 | Ankyrin | *Fjoh_0898* |
| A5FN42 | 55.11 | 6.14 | Nucleotidyl transferase | *Fjoh_0347* |
| A5FFQ0 | 25.06 | 6.09 | Uncharacterized protein | *Fjoh_2946* |
| A5FGN8 | 22.26 | 6.03 | Orotate phosphoribyltransferase | *Fjoh_2604* |
| A5FNZ0 | 30.81 | 6.01 | Queuine tRNA-ribyltransferase | *Fjoh_0045* |
| A5FA57 | 59.63 | 5.96 | Signal transduction histidine kinase, LytS | *Fjoh_4913* |
| A5FJ86 | 25.01 | 5.93 | Trigger factor, N-terminal domain protein | *Fjoh_1701* |
| A5FA44 | 34.98 | 5.73 | Uncharacterized protein | *Fjoh_4922* |
| A5FGW8 | 50.63 | 5.57 | Putative PAS/PAC sensor protein | *Fjoh_2525* |
| A5FI81 | 28.83 | 5.29 | Uncharacterized protein | *Fjoh_2057* |
| A5FM23 | 40.88 | 5.22 | ATPase associated with various cellular activities, AAA_3 | *Fjoh_0715* |
| A5FBN9 | 46.08 | 5.2 | Uncharacterized protein | *Fjoh_4365* |
| A5FJW1 | 29.33 | 5.09 | Electron transfer flavoprotein beta-subunit | *Fjoh_1474* |
| A5FLX6 | 69.03 | 4.99 | Uncharacterized protein | *Fjoh_0767* |
| A5FKV8 | 31.2 | 4.88 | PfkB domain carbohydrate kinase | *Fjoh_1131* |
| A5FIX8 | 52.04 | 4.87 | Peptidoglycan glycosyltransferase | *Fjoh_1805* |
| A5FGN0 | 27.46 | 4.84 | PhnA protein-like protein | *Fjoh_2615* |
| A5FB50 | 67.72 | 4.68 | Uncharacterized protein | *Fjoh_4571* |
| A5FMZ8 | 34.59 | 4.62 | 50S ribosomal protein L23 | *Fjoh_0395* |
| A5FNC1 | 30.33 | 4.48 | Uncharacterized protein | *Fjoh_0265* |
| A5FFX9 | 31.33 | 4.47 | Histidine biosynthesis bifunctional protein HisB | *Fjoh_2875* |
| A5FHB8 | 51.78 | 4.37 | Glucosamine-6-phosphate isomerase | *Fjoh_2381* |
| A5FM12 | 59.24 | 4.33 | BatB-like protein | *Fjoh_0720* |
| A5FNE1 | 35.7 | 4.33 | Uncharacterized protein | *Fjoh_0241* |
| A5FEB1 | 42.04 | 4.28 | N-acetyltransferase domain-containing protein | *Fjoh_3436* |
| A5FEC5 | 50.95 | 4.24 | Aminotransferase | *Fjoh_3433* |
| A5FN09 | 41.07 | 4.19 | Uncharacterized protein containing a pentein-type domain-like protein | *Fjoh_0365* |
| A5FJ95 | 33.45 | 4.03 | Pyridoxine 5'-phosphate synthase | *Fjoh_1695* |
| A5FFZ2 | 23.6 | 4.02 | FAD-dependent pyridine nucleotide-disulphide oxidoreductase | *Fjoh_2851* |
| A5FFY0 | 45.27 | 3.95 | Histidinol-phosphate aminotransferase | *Fjoh_2876* |
| A5FIR9 | 51.61 | 3.92 | ABC transporter related | *Fjoh_1860* |
| A5FK92 | 34.07 | 3.89 | Ammonium transporter | *Fjoh_1337* |
| A5FJ40 | 31.43 | 3.85 | Aminotransferase | *Fjoh_1747* |
| A5FHX6 | 60.19 | 3.84 | Glutamate synthase (NADH) small subunit | *Fjoh_2160* |
| A5FK72 | 35.98 | 3.81 | bPH_3 domain-containing protein | *Fjoh_1370* |
| A5F9R5 | 44.14 | 3.79 | Histidine--tRNA ligase | *Fjoh_5052* |
| A5FB24 | 39.12 | 3.76 | Glyceraldehyde-3-phosphate dehydrogenase | *Fjoh_4590* |
| A5F9U6 | 34.18 | 3.72 | Sulfatase | *Fjoh_5023* |
| A5FM11 | 48.41 | 3.66 | BatA-like protein | *Fjoh_0719* |
| A5FM89 | 34.04 | 3.63 | 3-oxoacyl-[acyl-carrier-protein] synthase 3 | *Fjoh_0646* |
| A5FGN7 | 25.37 | 3.63 | Uncharacterized protein | *Fjoh_2603* |
| A5FKU8 | 21.05 | 3.52 | 3-oxoacyl-[acyl-carrier-protein] synthase 2 | *Fjoh_1138* |
| A5FN15 | 57.97 | 3.46 | DNA-directed RNA polymerase subunit alpha | *Fjoh_0371* |
| A5FIF4 | 39.97 | 3.42 | Luciferase family protein | *Fjoh_1983* |
| A5FGX2 | 48.72 | 3.41 | Probable malate:quinone oxidoreductase | *Fjoh_2529* |
| A5FIJ7 | 44.99 | 3.41 | Peptide chain release factor 3 | *Fjoh_1947* |
| A5FJ88 | 36 | 3.41 | ATP-dependent Clp protease ATP-binding subunit ClpX | *Fjoh_1703* |
| A5FN44 | 43.32 | 3.39 | GDP-L-fucose synthase | *Fjoh_0329* |
| A5FN02 | 27.93 | 3.35 | 50S ribosomal protein L15 | *Fjoh_0378* |
| A5FCI8 | 32.41 | 3.33 | Uncharacterized protein | *Fjoh_4073* |
| A5FJP8 | 39.64 | 3.33 | Uncharacterized protein | *Fjoh_1531* |
| A5FNT0 | 21.72 | 3.31 | 50S ribosomal protein L31 | *Fjoh_0109* |
| A5FFQ8 | 20.55 | 3.3 | Efflux transporter, RND family, MFP subunit | *Fjoh_2939* |
| A5FMS7 | 54.82 | 3.3 | MotA/TolQ/ExbB proton channel | *Fjoh_0460* |
| A5FKH4 | 26.98 | 3.29 | Transcriptional regulator, TetR family | *Fjoh_1265* |
| A5FME6 | 33.86 | 3.27 | Asparagine--tRNA ligase | *Fjoh_0583* |
| A5F9R9 | 31.58 | 3.23 | DEAD/DEAH box helicase domain protein | *Fjoh_5046* |
| A5FCM4 | 25.49 | 3.18 | PfkB domain protein | *Fjoh_4043* |
| A5FIJ5 | 34.26 | 3.15 | Uncharacterized protein | *Fjoh_1945* |
| A5FLQ5 | 25.07 | 3.15 | TM2 domain containing protein+B7201 | *Fjoh_0837* |
| A5FNC2 | 31.03 | 3.14 | Mannose-6-phosphate isomerase | *Fjoh_0266* |
| A5FEC6 | 34.12 | 3.13 | N-acetyl-gamma-glutamyl-phosphate reductase | *Fjoh_3434* |
| A5FNS3 | 42.91 | 3.13 | Peptidase, family S33 unassigned peptidases | *Fjoh_0102* |
| A5FA46 | 38.63 | 3.11 | Uncharacterized protein | *Fjoh_4924* |
| A5FM24 | 42.89 | 3.1 | VWFA domain-containing protein | *Fjoh_0716* |
| A5FIQ9 | 39.26 | 3.08 | GTPase Era | *Fjoh_1878* |
| A5FCA6 | 26.93 | 3.04 | Uncharacterized protein | *Fjoh_4154* |
| A5FA32 | 52.48 | 3.03 | 50S ribosomal subunit assembly factor BipA | *Fjoh_4935* |
| A5FGV5 | 51.86 | 2.98 | Peptide deformylase | *Fjoh_2532* |
| A5FN61 | 40.13 | 2.98 | Transport permease protein | *Fjoh_0327* |
| A5FKH6 | 37.8 | 2.97 | Efflux transporter, RND family, MFP subunit | *Fjoh_1267* |
| A5FN27 | 25.64 | 2.9 | Nucleotidyl transferase | *Fjoh_0349* |
| A5FH20 | 60.6 | 2.89 | Ribosome-binding ATPase YchF | *Fjoh_2467* |
| A5FJ26 | 47.65 | 2.87 | 2,3-bisphosphoglycerate-independent phosphoglycerate mutase | *Fjoh_1764* |
| A5FG81 | 32.52 | 2.85 | 30S ribosomal protein S1 | *Fjoh_2762* |
| A5FJI5 | 41.41 | 2.85 | Uncharacterized protein | *Fjoh_1594* |
| A5FN20 | 42.13 | 2.84 | UDP-glucose 6-dehydrogenase | *Fjoh_0358* |
| A5FN29 | 28.27 | 2.84 | Uncharacterized protein | *Fjoh_0351* |
| A5FA39 | 47.29 | 2.83 | Uncharacterized protein | *Fjoh_4933* |
| A5FGN3 | 26.48 | 2.82 | D-alanine--D-alanine ligase | *Fjoh_2618* |
| A5FGC2 | 53.1 | 2.8 | Heavy metal efflux pump, CzcA family | *Fjoh_2731* |
| A5FCT0 | 20.18 | 2.79 | Binding-protein-dependent transport systems inner membrane component | *Fjoh_3985* |
| A5FFU4 | 30.59 | 2.78 | Response regulator receiver protein | *Fjoh_2906* |
| A5FIK4 | 47.67 | 2.78 | Cystathionine beta-synthase | *Fjoh_1927* |
| A5FFY5 | 58.46 | 2.76 | Acetolactate synthase | *Fjoh_2862* |
| A5FMM0 | 26.06 | 2.76 | Ribonucleoside-diphosphate reductase | *Fjoh_0521* |
| A5FLG5 | 25.28 | 2.72 | Uncharacterized protein | *Fjoh_0924* |
| A5FAU1 | 32.17 | 2.7 | NUMOD4 domain protein | *Fjoh_4675* |
| A5FJU6 | 29.71 | 2.7 | Aspartate kinase | *Fjoh_1494* |
| A5FLS1 | 25.02 | 2.69 | ATP synthase subunit beta | *Fjoh_0819* |
| A5FL33 | 31.4 | 2.68 | ATP synthase subunit delta | *Fjoh_1058* |
| A5FNF9 | 41.85 | 2.68 | Lipoyl synthase | *Fjoh_0227* |
| A5FLI9 | 41.14 | 2.67 | Ankyrin | *Fjoh_0897* |
| A5FMM2 | 35.33 | 2.67 | Uncharacterized protein | *Fjoh_0504* |
| A5FJB5 | 30.67 | 2.65 | MscS Mechanosensitive ion channel | *Fjoh_1674* |
| A5FK52 | 47.59 | 2.65 | Ribosomal large subunit pseudouridine synthase B | *Fjoh_1384* |
| A5FFN6 | 33.44 | 2.64 | SSS sodium solute transporter superfamily | *Fjoh_2962* |
| A5FME8 | 34.83 | 2.63 | Uncharacterized protein | *Fjoh_0585* |
| A5FKQ8 | 35.66 | 2.61 | FeS assembly protein SufB | *Fjoh_1184* |
| A5F9S3 | 39.55 | 2.59 | Chaperone protein DnaJ | *Fjoh_5050* |
| A5FGV6 | 36.89 | 2.59 | DUF5606 domain-containing protein | *Fjoh_2533* |
| A5FHP0 | 32.55 | 2.57 | Asparagine synthase (Glutamine-hydrolyzing) | *Fjoh_2252* |
| A5FNX7 | 35.01 | 2.56 | Transketolase domain protein | *Fjoh_0048* |
| A5FHI8 | 31.26 | 2.55 | Peptide chain release factor 1 | *Fjoh_2309* |
| A5FM99 | 35.5 | 2.55 | Two component, sigma54 specific, transcriptional regulator, Fis family | *Fjoh_0638* |
| A5FNS4 | 22.54 | 2.55 | N-acetyltransferase domain-containing protein | *Fjoh_0103* |
| A5FEW6 | 23.62 | 2.53 | Transporter, hydrophobe/amphiphile efflux-1 (HAE1) family | *Fjoh_3227* |
| A5FE44 | 40.22 | 2.53 | Triosephosphate isomerase | *Fjoh_3504* |
| A5FBY4 | 39.06 | 2.52 | Acriflavin resistance protein | *Fjoh_4285* |
| A5FKQ4 | 31.49 | 2.52 | FeS assembly protein SufD | *Fjoh_1180* |
| A5FMU9 | 43.44 | 2.52 | Peptidase family M3 Oligopeptidase A | *Fjoh_0429* |
| A5FN91 | 39.49 | 2.52 | ABC transporter related | *Fjoh_0285* |
| A5FND0 | 36.54 | 2.52 | Heat shock protein Hsp90 | *Fjoh_0260* |
| A5FGQ7 | 30.83 | 2.51 | Aminotransferase | *Fjoh_2591* |
| A5FL35 | 24.74 | 2.49 | Uncharacterized protein | *Fjoh_1045* |
| A5FJB7 | 46.45 | 2.48 | Nitrogen-fixing NifU domain protein | *Fjoh_1676* |
| A5FJG5 | 37.69 | 2.48 | Metallophosphoesterase | *Fjoh_1623* |
| A5FP02 | 49.39 | 2.48 | Bacterial translation initiation factor 3 (BIF-3) | *Fjoh_0025* |
| A5FC55 | 35.45 | 2.47 | Transcriptional regulator, XRE family | *Fjoh_4208* |
| A5FKC6 | 43.62 | 2.47 | 3-isopropylmalate dehydratase large subunit | *Fjoh_1307* |
| A5FNY7 | 57.19 | 2.46 | Acetyl-coenzyme A carboxylase carboxyl transferase subunit alpha | *Fjoh_0042* |
| A5FB75 | 20.8 | 2.45 | Allergen V5/Tpx-1 family protein | *Fjoh_4541* |
| A5FM91 | 32.07 | 2.45 | Uncharacterized protein | *Fjoh_0648* |
| A5F9T4 | 21.65 | 2.44 | Uncharacterized protein | *Fjoh_5038* |
| A5FHN9 | 23.71 | 2.44 | Asparagine synthase (Glutamine-hydrolyzing) | *Fjoh_2251* |
| A5FNU8 | 41.07 | 2.44 | Putative tRNA (cytidine(34)-2'-O)-methyltransferase | *Fjoh_0078* |
| A5FJP6 | 27.02 | 2.43 | 50S ribosomal protein L27 | *Fjoh_1546* |
| A5FNN7 | 30.94 | 2.43 | Methionyl-tRNA formyltransferase | *Fjoh_0138* |
| A5FA38 | 35.17 | 2.42 | ABC transporter related | *Fjoh_4932* |
| A5FDK7 | 31.47 | 2.42 | Uncharacterized protein | *Fjoh_3707* |
| A5FN33 | 40.73 | 2.42 | Mannose-1-phosphate guanylyltransferase (GDP) | *Fjoh_0355* |
| A5FLJ7 | 22.52 | 2.41 | 3-oxoacid CoA-transferase, A subunit | *Fjoh_0888* |
| A5FHA4 | 26.14 | 2.4 | CTP synthase | *Fjoh_2383* |
| A5FMM5 | 25.21 | 2.4 | S-adenosylmethionine:tRNA ribosyltransferase-isomerase | *Fjoh_0507* |
| A5FNH3 | 20.16 | 2.4 | 6,7-dimethyl-8-ribityllumazine synthase | *Fjoh_0212* |
| A5FL84 | 23.64 | 2.39 | Methyltransferase type 11 | *Fjoh_1001* |
| A5FAH4 | 26.25 | 2.37 | Glucose-6-phosphate 1-dehydrogenase | *Fjoh_4800* |
| A5FM06 | 30.86 | 2.36 | 4-hydroxyphenylpyruvate dioxygenase | *Fjoh_0729* |
| A5FIL3 | 36.92 | 2.35 | Lysine--tRNA ligase | *Fjoh_1919* |
| A5FJW0 | 31.84 | 2.35 | Electron transfer flavoprotein, alpha subunit | *Fjoh_1473* |
| A5FIT7 | 31.06 | 2.34 | Protein-export membrane protein SecG | *Fjoh_1845* |
| A5FFX5 | 33.43 | 2.33 | Histidine biosynthesis bifunctional protein HisIE | *Fjoh_2871* |
| A5FJF7 | 30.16 | 2.33 | Ribosome maturation factor RimP | *Fjoh_1628* |
| A5FJQ1 | 26.86 | 2.32 | Histone family protein DNA-binding protein | *Fjoh_1534* |
| A5FAP2 | 38.87 | 2.31 | Zinc finger, SWIM domain protein | *Fjoh_4732* |
| A5FK18 | 43.65 | 2.3 | PpiC-type peptidyl-prolyl cis-trans isomerase | *Fjoh_1415* |
| A5FDT3 | 36.49 | 2.3 | KAP P-loop domain protein | *Fjoh_3630* |
| A5FGC6 | 27.3 | 2.28 | Beta sliding clamp | *Fjoh_2720* |
| A5FNS2 | 37.83 | 2.27 | Uncharacterized protein | *Fjoh_0101* |
| A5FL66 | 30.01 | 2.26 | Uncharacterized protein | *Fjoh_1024* |
| A5FBD8 | 29.91 | 2.25 | Nitroreductase | *Fjoh_4487* |
| A5FIZ2 | 29.56 | 2.25 | Peptidase family S33 | *Fjoh_1801* |
| A5FEG6 | 32.28 | 2.24 | Predicted NADH:flavin oxidoreductase/NADH oxidase | *Fjoh_3385* |
| A5FGU1 | 47.4 | 2.23 | Oligopeptidase B | *Fjoh_2556* |
| A5FIV1 | 24.72 | 2.23 | Chaperonin GroEL | *Fjoh_1842* |
| A5FMG4 | 31.33 | 2.22 | Cell division protein FtsX | *Fjoh_0567* |
| A5FNI6 | 39.85 | 2.22 | Thioredoxin reductase | *Fjoh_0199* |
| A5FL79 | 25.01 | 2.21 | Glutamine--tRNA ligase | *Fjoh_1012* |
| A5FN00 | 39.08 | 2.21 | Translation initiation factor IF-1 | *Fjoh_0376* |
| A5FJM6 | 34.47 | 2.2 | Uncharacterized protein | *Fjoh_1568* |
| A5FJZ2 | 30.63 | 2.2 | 30S ribosomal protein S15 | *Fjoh_1445* |
| A5FKW3 | 27.95 | 2.2 | L-arabinose isomerase | *Fjoh_1121* |
| A5FLE7 | 24.81 | 2.2 | Uroporphyrinogen decarboxylase | *Fjoh_0946* |
| A5F9Y4 | 25.39 | 2.19 | HPt domain-containing protein | *Fjoh_4985* |
| A5FB85 | 36.74 | 2.19 | Putative phosphohistidine phosphatase, SixA | *Fjoh_4536* |
| A5FL67 | 42.8 | 2.19 | GH3 auxin-responsive promoter | *Fjoh_1025* |
| A5FB31 | 41.27 | 2.18 | Mammalian cell entry related domain protein | *Fjoh_4584* |
| A5FNP8 | 34.54 | 2.18 | UDP-N-acetylglucosamine 1-carboxyvinyltransferase | *Fjoh_0131* |
| A5FBB5 | 40.79 | 2.16 | Cytokinin riboside 5'-monophosphate phosphoribohydrolase | *Fjoh_4510* |
| A5FK21 | 28.67 | 2.16 | 3-hydroxy-3-methylglutaryl coenzyme A reductase | *Fjoh_1418* |
| A5FE92 | 43.77 | 2.15 | DNA repair protein RecN | *Fjoh_3465* |
| A5FE94 | 28.11 | 2.15 | Coenzyme A biosynthesis bifunctional protein CoaBC | *Fjoh_3467* |
| A5FJT6 | 26.62 | 2.15 | Sulfite reductase (Ferredoxin) | *Fjoh_1504* |
| A5FNT7 | 21.32 | 2.14 | Carbamoyl-phosphate synthase (glutamine-hydrolyzing) | *Fjoh_0099* |
| A5FHC3 | 24.78 | 2.14 | Cl-channel, voltage-gated family protein | *Fjoh_2371* |
| A5FIU0 | 22.4 | 2.14 | Transcriptional regulator | *Fjoh_1848* |
| A5FC70 | 22.81 | 2.13 | Short-chain dehydrogenase/reductase SDR | *Fjoh_4190* |
| A5FL10 | 43.69 | 2.13 | Ribosome-binding factor A | *Fjoh_1069* |
| A5FHC6 | 42.12 | 2.11 | Uncharacterized protein | *Fjoh_2359* |
| A5FIK1 | 22.52 | 2.11 | Transcription termination/antitermination protein NusG | *Fjoh_1938* |
| A5FML3 | 20.82 | 2.1 | DAHP synthetase I/KDSA | *Fjoh_0514* |
| A5FN39 | 23.5 | 2.1 | Uncharacterized protein | *Fjoh_0344* |
| A5FK53 | 28.76 | 2.09 | UbiA prenyltransferase | *Fjoh_1385* |
| A5FKV3 | 31.83 | 2.09 | Ribose-phosphate diphosphokinase | *Fjoh_1126* |
| A5FLA2 | 30.38 | 2.08 | Protein RecA | *Fjoh_0988* |
| A5FBR5 | 42.17 | 2.07 | Heat shock protein DnaJ domain protein | *Fjoh_4342* |
| A5FEN1 | 33.38 | 2.07 | Luciferase family protein | *Fjoh_3319* |
| A5FJI4 | 36.35 | 2.07 | Elongation factor Ts | *Fjoh_1609* |
| A5FMT5 | 39.63 | 2.07 | ABC transporter related | *Fjoh_0450* |
| A5FNH8 | 42.55 | 2.07 | ParB-like partition protein | *Fjoh_0203* |
| A5FG89 | 28.7 | 2.06 | Lon protease | *Fjoh_2754* |
| A5FHA9 | 56.83 | 2.06 | tRNA-specific 2-thiouridylase MnmA | *Fjoh_2388* |
| A5FB65 | 26.87 | 2.05 | Glucosamine-6-phosphate deaminase | *Fjoh_4557* |
| A5FEX0 | 24.73 | 2.05 | 3-oxoacyl-[acyl-carrier-protein] synthase 3 | *Fjoh_3231* |
| A5FHA5 | 36.64 | 2.05 | Membrane protein insertase YidC | *Fjoh_2384* |
| A5FNA5 | 22.43 | 2.05 | KpsF/GutQ family protein | *Fjoh_0282* |
| A5FGX5 | 26.8 | 2.04 | Candidate alpha-glycosyltransferase Glycosyltransferase family 4 | *Fjoh_2513* |
| A5FJ47 | 44.87 | 2.04 | Cyclic nucleotide-binding protein | *Fjoh_1740* |
| A5FKQ6 | 25.76 | 2.04 | FeS assembly ATPase SufC | *Fjoh_1182* |
| A5FBS6 | 34.7 | 2.03 | Uncharacterized protein with a von Willebrand factor type A (VWA) domain-like protein | *Fjoh_4337* |
| A5FGC7 | 50.98 | 2.03 | GldG | *Fjoh_2721* |
| A5FNF0 | 21.5 | 2.03 | Uncharacterized protein | *Fjoh_0234* |
| A5FAK5 | 20.04 | 2.02 | Branched-chain-amino-acid aminotransferase | *Fjoh_4770* |
| A5FH79 | 20.85 | 2.02 | Ferredoxin-dependent glutamate synthase | *Fjoh_2409* |
| A5FHV7 | 27.95 | 2.01 | tRNA (guanine-N(1)-)-methyltransferase | *Fjoh_2184* |
| A5FCT7 | 22.33 | 2 | Major facilitator superfamily MFS_1 | *Fjoh_3965* |
| A5FKS9 | 52.38 | 2 | Zinc metalloprotease | *Fjoh_1159* |
| A5FM08 | 20.43 | 2 | Tryptophan 2 3-dioxygenase (Vermilion)-like protein | *Fjoh_0731* |
| A5FMX0 | 31.41 | 2 | GTPase Obg | *Fjoh_0418* |
| A5FNA8 | 44.56 | 1.99 | Alkyl hydroperoxide reductase/ Thiol specific antioxidant/ Mal allergen | *Fjoh_0267* |
| A5FCR6 | 29.26 | 1.98 | ABC transporter, ATPase subunit | *Fjoh_3986* |
| A5FEC0 | 36.06 | 1.98 | Peptidase family M20 | *Fjoh_3428* |
| A5FG70 | 27.66 | 1.98 | 1,4-dihydroxy-2-naphthoyl-CoA synthase | *Fjoh_2784* |
| A5FMP2 | 26.1 | 1.98 | Uncharacterized protein | *Fjoh_0491* |
| A5FMM4 | 24.43 | 1.97 | Kynureninase | *Fjoh_0506* |
| A5FEK4 | 20.07 | 1.96 | Histidine kinase | *Fjoh_3354* |
| A5FHC8 | 22.67 | 1.96 | Uncharacterized protein | *Fjoh_2361* |
| A5FL34 | 38.47 | 1.96 | ATP synthase subunit alpha | *Fjoh_1059* |
| A5FFY2 | 25.19 | 1.95 | Acetyl-coenzyme A synthetase | *Fjoh_2859* |
| A5FJR5 | 29.19 | 1.95 | GldA | *Fjoh_1516* |
| A5FNG4 | 20.14 | 1.95 | UDP-N-acetylenolpyruvoylglucosamine reductase | *Fjoh_0218* |
| A5FFQ9 | 41.1 | 1.95 | Acriflavin resistance protein | *Fjoh_2940* |
| A5FAI5 | 28.73 | 1.93 | Probable potassium transport system protein kup | *Fjoh_4780* |
| A5FB71 | 26.56 | 1.93 | ABC transporter related | *Fjoh_4550* |
| A5FHT2 | 26.36 | 1.93 | Dihydroorotate dehydrogenase (quinone) | *Fjoh_2210* |
| A5FJH6 | 38 | 1.92 | DNA polymerase I | *Fjoh_1617* |
| A5FIU2 | 40.09 | 1.92 | tRNA-2-methylthio-N(6)-dimethylallyladenosine synthase | *Fjoh_1850* |
| A5FGR2 | 24.84 | 1.91 | Phosphate transporter | *Fjoh_2578* |
| A5F9X8 | 30.79 | 1.9 | Dihydrolipoamide acetyltransferase component of pyruvate dehydrogenase complex | *Fjoh_4988* |
| A5FII6 | 25.74 | 1.9 | ATPase associated with various cellular activities, AAA_3 | *Fjoh_1950* |
| A5FJ54 | 24.58 | 1.9 | Malonyl CoA-acyl carrier protein transacylase | *Fjoh_1730* |
| A5FE75 | 24.7 | 1.88 | 50S ribosomal protein L9 | *Fjoh_3480* |
| A5FGR7 | 31.84 | 1.88 | ATPase AAA-2 domain protein | *Fjoh_2583* |
| A5FKJ2 | 24.71 | 1.88 | Aspartate--tRNA ligase | *Fjoh_1248* |
| A5FEC2 | 24.63 | 1.87 | N-succinylornithine carbamoyltransferase | *Fjoh_3430* |
| A5FE77 | 25.23 | 1.87 | 30S ribosomal protein S6 | *Fjoh_3482* |
| A5FIT4 | 39.85 | 1.87 | Uncharacterized protein | *Fjoh_1859* |
| A5FN28 | 30.56 | 1.86 | HAD-superfamily hydrolase, subfamily IA, variant 3 | *Fjoh_0350* |
| A5FL86 | 50.15 | 1.85 | tRNA uridine 5-carboxymethylaminomethyl modification enzyme MnmG | *Fjoh_1003* |
| A5FN53 | 30.13 | 1.85 | Alpha-glycosyltransferase-like protein Glycosyltransferase family 4 | *Fjoh_0338* |
| A5FC40 | 28.7 | 1.84 | Uncharacterized protein | *Fjoh_4227* |
| A5FK98 | 28.43 | 1.84 | Thioredoxin | *Fjoh_1343* |
| A5FMM1 | 28.07 | 1.84 | Ribonucleoside-diphosphate reductase | *Fjoh_0522* |
| A5FJ11 | 21.1 | 1.83 | Sec-independent translocation protein mttA/Hcf106 | *Fjoh_1774* |
| A5FM88 | 23.6 | 1.83 | Biotin carboxyl carrier protein of acetyl-CoA carboxylase | *Fjoh_0645* |
| A5FAW0 | 27.5 | 1.82 | Transcription termination factor Rho | *Fjoh_4662* |
| A5FH29 | 31.45 | 1.82 | Uncharacterized protein | *Fjoh_2463* |
| A5FGP8 | 23.8 | 1.81 | Phosphatidylserine decarboxylase related protein | *Fjoh_2597* |
| A5FIK7 | 35.41 | 1.81 | Amino acid permease-associated region | *Fjoh_1930* |
| A5FKK0 | 26.64 | 1.81 | NADH-quinone oxidoreductase subunit H | *Fjoh_1239* |
| A5FKV5 | 38 | 1.81 | Peptidyl-tRNA hydrolase | *Fjoh_1128* |
| A5FL73 | 28.15 | 1.81 | Glutamate--tRNA ligase | *Fjoh_1006* |
| A5FLR3 | 29.19 | 1.81 | DNA repair protein RadA | *Fjoh_0828* |
| A5FKI2 | 24.7 | 1.79 | Oxoglutarate dehydrogenase (succinyl-transferring) | *Fjoh_1256* |
| A5FGS9 | 26.2 | 1.79 | Aminomethyltransferase | *Fjoh_2562* |
| A5FKK1 | 28.47 | 1.79 | NADH dehydrogenase subunit G | *Fjoh_1240* |
| A5FAR1 | 24.7 | 1.78 | Candidate deacetylase Carbohydrate esterase family 14 | *Fjoh_4717* |
| A5FEM9 | 22.22 | 1.78 | Probable membrane transporter protein | *Fjoh_3317* |
| A5FJV8 | 31.97 | 1.78 | Thymidylate synthase | *Fjoh_1471* |
| A5FA80 | 28.75 | 1.77 | Nitroreductase | *Fjoh_4887* |
| A5FAL3 | 23.28 | 1.77 | Carbonic anhydrase | *Fjoh_4763* |
| A5FNW1 | 25.13 | 1.77 | Short-chain dehydrogenase/reductase SDR | *Fjoh_0075* |
| A5F9W1 | 56.89 | 1.76 | tRNA (guanosine(18)-2'-O)-methyltransferase | *Fjoh_5009* |
| A5FHY9 | 23.55 | 1.76 | Diaminopimelate decarboxylase | *Fjoh_2159* |
| A5FM21 | 37.55 | 1.76 | Short-chain dehydrogenase/reductase SDR | *Fjoh_0713* |
| A5FN34 | 24.32 | 1.76 | UDP-glucose/GDP-mannose dehydrogenase | *Fjoh_0356* |
| A5FKD9 | 24.83 | 1.75 | 30S ribosomal protein S16 | *Fjoh_1301* |
| A5FMY2 | 29.72 | 1.75 | 30S ribosomal protein S10 | *Fjoh_0398* |
| A5FJH1 | 29.04 | 1.74 | 50S ribosomal protein L13 | *Fjoh_1612* |
| A5FLK4 | 20.05 | 1.73 | Nucleoside recognition domain protein | *Fjoh_0879* |
| A5FLZ1 | 20.14 | 1.73 | Nicotinamide phosphoribosyltransferase | *Fjoh_0745* |
| A5F9T2 | 21.03 | 1.72 | Scramblase family protein | *Fjoh_5036* |
| A5FH66 | 21.28 | 1.72 | Polysaccharide biosynthesis protein | *Fjoh_2426* |
| A5FHP2 | 21.34 | 1.72 | DNA gyrase subunit B | *Fjoh_2254* |
| A5FHQ2 | 23.09 | 1.72 | ABC transporter related | *Fjoh_2245* |
| A5FJ52 | 28.18 | 1.72 | UDP-glucose 4-epimerase | *Fjoh_1728* |
| A5FNH4 | 25.74 | 1.71 | Tetratricopeptide TPR_2 repeat protein | *Fjoh_0213* |
| A5FA65 | 25.44 | 1.7 | Secretion protein HlyD family protein | *Fjoh_4903* |
| A5FKH7 | 27.71 | 1.7 | Acriflavin resistance protein | *Fjoh_1268* |
| A5FA68 | 20.19 | 1.69 | Polynucleotide adenylyltransferase/metal dependent phosphohydrolase | *Fjoh_4906* |
| A5FH63 | 25.24 | 1.69 | Uncharacterized protein | *Fjoh_2423* |
| A5FJB9 | 25.95 | 1.69 | Uncharacterized protein | *Fjoh_1662* |
| A5FMT3 | 20.3 | 1.68 | Candidate beta-glycosyltransferase Glycosyltransferase family 2 | *Fjoh_0448* |
| A5FFT5 | 35.1 | 1.67 | Energy-dependent translational throttle protein EttA | *Fjoh_2916* |
| A5FIZ1 | 26.46 | 1.67 | Probable GTP-binding protein EngB | *Fjoh_1800* |
| A5FH80 | 52.47 | 1.66 | HTHT-type domain-containing protein | *Fjoh_2410* |
| A5FJP9 | 28.58 | 1.66 | Ribonuclease, Rne/Rng family | *Fjoh_1532* |
| A5FK09 | 21.65 | 1.65 | Amino acid/peptide transporter | *Fjoh_1421* |
| A5FNA0 | 22.28 | 1.65 | Alcohol dehydrogenase, zinc-binding domain protein | *Fjoh_0277* |
| A5FLU2 | 48.77 | 1.64 | Glycine--tRNA ligase | *Fjoh_0796* |
| A5FMY0 | 23.46 | 1.64 | 50S ribosomal protein L4 | *Fjoh_0396* |
| A5FJ56 | 29.24 | 1.63 | Transcriptional regulator, AraC family | *Fjoh_1732* |
| A5FKJ0 | 23.98 | 1.62 | NADH-quinone oxidoreductase subunit A | *Fjoh_1246* |
| A5FNX8 | 42.15 | 1.61 | Transketolase, central region | *Fjoh_0049* |
| A5F9Y8 | 20.59 | 1.61 | Signal recognition particle receptor FtsY | *Fjoh_4980* |
| A5FJN0 | 26.27 | 1.61 | UDP-N-acetylmuramoyl-tripeptide--D-alanyl-D-alanine ligase | *Fjoh_1558* |
| A5FB09 | 26.37 | 1.6 | NLP/P60 protein dipeptidyl peptidase VI | *Fjoh_4613* |
| A5FNH7 | 50.2 | 1.6 | Cobyrinic acid a,c-diamide synthase | *Fjoh_0202* |
| A5FLP8 | 20.74 | 1.59 | Aldo/keto reductase | *Fjoh_0830* |
| A5FIP9 | 34.35 | 1.59 | Alanine--tRNA ligase | *Fjoh_1894* |
| A5FN47 | 22.33 | 1.58 | dTDP-4-dehydrorhamnose reductase | *Fjoh_0332* |
| A5F9U9 | 36.01 | 1.57 | Peroxidase | *Fjoh_5017* |
| A5FEA5 | 21.19 | 1.57 | Protein-L-isoaspartate O-methyltransferase | *Fjoh_3446* |
| A5FF61 | 23.22 | 1.57 | Amino acid permease-associated region | *Fjoh_3147* |
| A5FJP5 | 21.93 | 1.57 | 50S ribosomal protein L21 | *Fjoh_1545* |
| A5FJT4 | 22.53 | 1.57 | Sulfate adenylyltransferase subunit 2 | *Fjoh_1502* |
| A5FLQ0 | 34.48 | 1.57 | Exodeoxyribonuclease III | *Fjoh_0832* |
| A5FNY9 | 27.26 | 1.57 | Permease YjgP/YjgQ family protein | *Fjoh_0044* |
| A5FK47 | 32.34 | 1.56 | HI0933 family protein | *Fjoh_1396* |
| A5FKI8 | 35.41 | 1.55 | NADH-quinone oxidoreductase subunit C | *Fjoh_1244* |
| A5FN30 | 42.64 | 1.55 | Lipopolysaccharide biosynthesis protein | *Fjoh_0352* |
| A5FJF9 | 35.18 | 1.54 | Translation initiation factor IF-2 | *Fjoh_1630* |
| A5FLK0 | 20.18 | 1.53 | PSP1 domain protein | *Fjoh_0891* |
| A5FBX9 | 20.95 | 1.52 | Short-chain dehydrogenase/reductase SDR | *Fjoh_4280* |
| A5FP20 | 27.79 | 1.52 | Methylmalonyl-CoA mutase-like protein | *Fjoh_0013* |
| A5FG35 | 21.14 | 1.51 | Deoxyhypusine synthase | *Fjoh_2810* |
| A5FMP1 | 27.01 | 1.51 | TonB family protein | *Fjoh_0490* |
| A5FHR9 | 24.77 | 0.66 | DUF58 domain-containing protein | *Fjoh_2227* |
| A5FGU5 | 31.04 | 0.65 | DsbD_2 domain-containing protein | *Fjoh_2541* |
| A5FGV8 | 27.68 | 0.65 | Putative transcriptional regulator, Crp/Fnr family | *Fjoh_2535* |
| A5FNK3 | 24.14 | 0.64 | Transcriptional regulator, AraC family | *Fjoh_0187* |
| A5FLW8 | 23.64 | 0.62 | Transcriptional regulator, LysR family | *Fjoh_0758* |
| A5FNX2 | 24.39 | 0.62 | Phytoene dehydrogenase-related protein | *Fjoh_0057* |
| A5FAH7 | 21.32 | 0.61 | Saccharopine dehydrogenase [NAD(+), L-lysine-forming] | *Fjoh_4787* |
| A5FD70 | 23.34 | 0.61 | Sodium/hydrogen exchanger | *Fjoh_3830* |
| A5FK90 | 26.42 | 0.61 | DUF58 domain-containing protein | *Fjoh_1349* |
| A5FFT4 | 24.3 | 0.6 | Putative thiol-disulphide oxidoreductase DCC | *Fjoh_2915* |
| A5FGV9 | 24.03 | 0.6 | Copper-exporting ATPase | *Fjoh_2536* |
| A5FH65 | 26.01 | 0.6 | Alpha-glycosyltransferase-like protein Glycosyltransferase family 4 | *Fjoh_2425* |
| A5FEA4 | 39.66 | 0.59 | Helix-turn-helix domain protein | *Fjoh_3445* |
| A5FG09 | 33.91 | 0.59 | ABC-type nitrate/sulfonate/bicarbonate transport systems periplasmic components-like protein | *Fjoh_2834* |
| A5FM38 | 20.48 | 0.59 | PDDEXK_1 domain-containing protein | *Fjoh_0696* |
| A5FM95 | 29.79 | 0.59 | Biosynthetic peptidoglycan transglycosylase | *Fjoh_0634* |
| A5FBW9 | 47.99 | 0.58 | Uncharacterized protein | *Fjoh_4288* |
| A5FFA5 | 28.36 | 0.57 | Transcriptional regulator, AraC family | *Fjoh_3096* |
| A5FIM6 | 36.6 | 0.57 | D-isomer specific 2-hydroxyacid dehydrogenase, NAD-binding | *Fjoh_1917* |
| A5FB42 | 36.98 | 0.55 | Candidate alpha-glycosyltransferase Glycosyltransferase family 4 | *Fjoh_4580* |
| A5FE29 | 24.64 | 0.55 | Uncharacterized protein | *Fjoh_3533* |
| A5FG12 | 20.43 | 0.54 | Purine or other phosphorylase, family 1 | *Fjoh_2837* |
| A5FH25 | 23.11 | 0.52 | Nicotinate phosphoribosyltransferase | *Fjoh_2472* |
| A5FP28 | 44.64 | 0.52 | Chromosomal replication initiator protein DnaA | *Fjoh_0005* |
| A5FCK7 | 21.48 | 0.51 | CopD domain-containing protein | *Fjoh_4061* |
| A5FGR1 | 21.88 | 0.51 | Uncharacterized protein | *Fjoh_2577* |
| A5F9X4 | 28.63 | 0.5 | Protein-tyrosine-phosphatase | *Fjoh_4993* |
| A5FGE3 | 21.87 | 0.5 | Transcriptional regulator, LacI family | *Fjoh_2710* |
| A5FC66 | 29.65 | 0.49 | (2Fe-2S)-binding domain protein | *Fjoh_4202* |
| A5FM03 | 21.14 | 0.49 | UvrABC system protein C | *Fjoh_0726* |
| A5FNY1 | 28.7 | 0.49 | Phosphoribosyltransferase | *Fjoh_0052* |
| A5FIX9 | 24.58 | 0.48 | UDP-N-acetylmuramoyl-L-alanyl-D-glutamate--2,6-diaminopimelate ligase | *Fjoh_1806* |
| A5FGK6 | 42.29 | 0.48 | AAA ATPase, central domain protein | *Fjoh_2640* |
| A5FIN8 | 25.36 | 0.47 | GSCFA domain-containing protein | *Fjoh_1897* |
| A5FB52 | 48.05 | 0.46 | DEAD/DEAH box helicase domain protein | *Fjoh_4573* |
| A5FMD7 | 28.65 | 0.46 | Peptidase family S33 | *Fjoh_0592* |
| A5FAK1 | 38.31 | 0.45 | Ferritin, Dps family protein | *Fjoh_4766* |
| A5FB05 | 24.13 | 0.45 | Uncharacterized protein | *Fjoh_4622* |
| A5FCX2 | 27.34 | 0.45 | NAD(P)-bd_dom domain-containing protein | *Fjoh_3938* |
| A5FCZ2 | 53.58 | 0.43 | Carbamoyltransferase | *Fjoh_3910* |
| A5FK97 | 22.95 | 0.43 | Hypothetical lipoprotein | *Fjoh_1342* |
| A5FL77 | 20.63 | 0.43 | Uncharacterized protein | *Fjoh_1010* |
| A5FA37 | 24.17 | 0.42 | Uncharacterized protein | *Fjoh_4931* |
| A5FC73 | 38.78 | 0.42 | Histidine kinase | *Fjoh_4193* |
| A5FE78 | 22.02 | 0.42 | Two component transcriptional regulator, LytTR family | *Fjoh_3483* |
| A5FA99 | 28.77 | 0.41 | Aldehyde dehydrogenase | *Fjoh_4869* |
| A5FB33 | 28.42 | 0.41 | Organic solvent tolerance protein OstA-like protein | *Fjoh_4586* |
| A5FGE2 | 29.7 | 0.41 | DNA mismatch repair protein MutS domain protein | *Fjoh_2709* |
| A5FLL7 | 23.75 | 0.41 | Haloacid dehalogenase domain protein hydrolase | *Fjoh_0862* |
| A5FNW5 | 24.95 | 0.41 | Uncharacterized protein | *Fjoh_0064* |
| A5FD03 | 21.08 | 0.4 | Hydrogenase maturation factor HypA | *Fjoh_3905* |
| A5FF42 | 35.33 | 0.4 | Peptidoglycan-binding domain 1 protein | *Fjoh_3167* |
| A5FMP5 | 22.92 | 0.4 | DUF853 domain-containing protein | *Fjoh_0494* |
| A5FHR5 | 44.32 | 0.39 | (P)ppGpp synthetase I, SpoT/RelA | *Fjoh_2223* |
| A5FIT3 | 35.62 | 0.39 | FAD dependent oxidoreductase | *Fjoh_1858* |
| A5FCK5 | 20.7 | 0.38 | Uncharacterized protein | *Fjoh_4059* |
| A5FC64 | 30.07 | 0.37 | Xanthine dehydrogenase, molybdenum binding subunit apoprotein | *Fjoh_4200* |
| A5FB89 | 42.26 | 0.37 | Uncharacterized protein | *Fjoh_4524* |
| A5FBE2 | 36.52 | 0.36 | Metal dependent phosphohydrolase | *Fjoh_4472* |
| A5FIS0 | 34.42 | 0.36 | DUF2183 domain-containing protein | *Fjoh_1861* |
| A5FAW8 | 36.98 | 0.35 | PCLP domain-containing protein | *Fjoh_4654* |
| A5FCZ4 | 53.01 | 0.35 | Hydrogenase expression/formation protein HypD | *Fjoh_3912* |
| A5FED6 | 44.77 | 0.35 | Argininosuccinate lyase | *Fjoh_3427* |
| A5FIX6 | 42.16 | 0.35 | Ribosomal RNA small subunit methyltransferase H | *Fjoh_1803* |
| A5FM68 | 29.4 | 0.35 | Uncharacterized protein | *Fjoh_0660* |
| A5FBB0 | 50.16 | 0.34 | Uncharacterized protein | *Fjoh_4505* |
| A5FE26 | 28.82 | 0.34 | Magnesium transport protein CorA | *Fjoh_3530* |
| A5FA02 | 38.11 | 0.33 | Beta-lactamase domain protein | *Fjoh_4971* |
| A5FGR6 | 48.35 | 0.33 | SSU ribosomal protein S6P modification protein | *Fjoh_2582* |
| A5FIW1 | 28.86 | 0.32 | S-adenosyl-L-methionine-dependent methyltransferase | *Fjoh_1821* |
| A5FJD4 | 33.13 | 0.32 | Uncharacterized protein | *Fjoh_1646* |
| A5FAW9 | 42.35 | 0.29 | Cysteine desulfurase | *Fjoh_4655* |
| A5FNS5 | 28.39 | 0.29 | Anion transporter | *Fjoh_0104* |
| A5FGP4 | 27.22 | 0.27 | Candidate alpha-glycosidase Glycoside hydrolase family 13 | *Fjoh_2593* |
| A5FL49 | 27.17 | 0.2 | Phosphoribosylamine--glycine ligase | *Fjoh_1041* |
| A5FAU5 | 26.06 | 0.19 | Uncharacterized protein | *Fjoh_4679* |
| A5FJ45 | 41.36 | 0.17 | Malate synthase | *Fjoh_1738* |
| A5FKE9 | 68.29 | 0.1 | VKc domain-containing protein | *Fjoh_1294* |
| A5FJ46 | 44.62 | 0.09 | Isocitrate lyase | *Fjoh_1739* |
| Δ*lolB1* | | | | |
| Accession | **Significance** | ***lolB1*/WT FC** | **Description** | **Gene code** |
| A5FA38 | 62.68 | 6.24 | ABC transporter related | *Fjoh_4932* |
| A5FA39 | 51.54 | 5.66 | Uncharacterized protein | *Fjoh_4933* |
| A5FFY5 | 20.89 | 43.78 | Acetolactate synthase | *Fjoh_2862* |
| A5FG23 | 21.48 | 3.70 | Uncharacterized protein | *Fjoh_2830* |
| A5FB75 | 29.75 | 3.38 | Allergen V5/Tpx-1 family protein | *Fjoh_4541* |
| A5FJP8 | 39.05 | 3.18 | Uncharacterized protein | *Fjoh_1531* |
| A5FBN9 | 42.58 | 3.11 | Uncharacterized protein | *Fjoh_4365* |
| A5FNS3 | 48.52 | 3.10 | Peptidase, family S33 unassigned peptidases | *Fjoh_0102* |
| A5FLJ0 | 43.28 | 2.87 | Ankyrin | *Fjoh_0898* |
| A5FMW3 | 22.77 | 2.41 | N5-carboxyaminoimidazole ribonucleotide mutase | *Fjoh_0426* |
| A5FMM2 | 43.91 | 2.33 | Uncharacterized protein | *Fjoh_0504* |
| A5FA57 | 23.94 | 2.22 | Signal transduction histidine kinase, LytS | *Fjoh_4913* |
| A5FB50 | 50.70 | 2.22 | Uncharacterized protein | *Fjoh_4571* |
| A5FAH3 | 27.34 | 2.19 | 6-phosphogluconate dehydrogenase, decarboxylating | *Fjoh_4799* |
| A5FLJ7 | 31.49 | 2.17 | 3-oxoacid CoA-transferase, A subunit | *Fjoh_0888* |
| A5FMS7 | 41.64 | 2.10 | MotA/TolQ/ExbB proton channel | *Fjoh_0460* |
| A5FNC2 | 27.57 | 2.08 | Mannose-6-phosphate isomerase | *Fjoh_0266* |
| A5FEG6 | 23.79 | 2.05 | Predicted NADH:flavin oxidoreductase/NADH oxidase | *Fjoh_3385* |
| A5FLX6 | 25.20 | 2.02 | Uncharacterized protein | *Fjoh_0767* |
| A5FGA5 | 26.77 | 1.98 | Uncharacterized protein | *Fjoh_2751* |
| A5FAW8 | 27.30 | 1.96 | PCLP domain-containing protein | *Fjoh_4654* |
| A5FM06 | 22.62 | 1.96 | 4-hydroxyphenylpyruvate dioxygenase | *Fjoh_0729* |
| A5FIP3 | 39.91 | 1.95 | Transcriptional regulator, BadM/Rrf2 family | *Fjoh_1888* |
| A5FAW9 | 32.19 | 1.93 | Cysteine desulfurase | *Fjoh_4655* |
| A5FJ54 | 22.45 | 1.84 | Malonyl CoA-acyl carrier protein transacylase | *Fjoh_1730* |
| A5FC55 | 33.40 | 1.82 | Transcriptional regulator, XRE family | *Fjoh_4208* |
| A5FGN3 | 23.95 | 1.80 | D-alanine--D-alanine ligase | *Fjoh_2618* |
| A5FKS2 | 21.68 | 1.78 | Oxidoreductase domain protein | *Fjoh_1166* |
| A5FG87 | 33.79 | 1.77 | ECF subfamily RNA polymerase sigma-24 subunit | *Fjoh_2752* |
| A5FFQ0 | 32.00 | 1.74 | Uncharacterized protein | *Fjoh_2946* |
| A5FFY0 | 25.03 | 1.74 | Histidinol-phosphate aminotransferase | *Fjoh_2876* |
| A5FIM7 | 23.10 | 1.64 | Aromatic amino acid hydroxylase | *Fjoh_1902* |
| A5FNI0 | 23.50 | 1.63 | Dihydrodipicolinate reductase | *Fjoh_0205* |
| A5FME6 | 21.01 | 1.63 | Asparagine--tRNA ligase | *Fjoh_0583* |
| A5FHB8 | 25.00 | 1.56 | Glucosamine-6-phosphate isomerase | *Fjoh_2381* |
| A5FM23 | 23.60 | 1.55 | ATPase associated with various cellular activities, AAA_3 | *Fjoh_0715* |
| A5FJ26 | 24.12 | 1.54 | 2,3-bisphosphoglycerate-independent phosphoglycerate mutase | *Fjoh_1764* |
| A5FE44 | 27.81 | 1.54 | Triosephosphate isomerase | *Fjoh_3504* |
| A5FHY9 | 23.77 | 1.51 | Diaminopimelate decarboxylase | *Fjoh_2159* |
| A5FHC3 | 20.93 | 0.66 | Cl-channel, voltage-gated family protein | *Fjoh_2371* |
| A5FKK7 | 25.59 | 0.66 | NADH-quinone oxidoreductase subunit N | *Fjoh_1233* |
| A5FFU3 | 21.67 | 0.65 | Metal dependent phosphohydrolase | *Fjoh_2905* |
| A5FG36 | 30.09 | 0.65 | Orn/DAP/Arg decarboxylase 2 | *Fjoh_2811* |
| A5FGT9 | 39.24 | 0.64 | Threonine aldolase | *Fjoh_2554* |
| A5FA90 | 21.97 | 0.63 | Cytochrome bd ubiquinol oxidase, subunit I | *Fjoh_4879* |
| A5FIX0 | 34.39 | 0.62 | Cell division protein FtsA | *Fjoh_1813* |
| A5FB33 | 46.47 | 0.61 | Organic solvent tolerance proteintA-like protein | *Fjoh_4586* |
| A5FIS9 | 32.23 | 0.59 | GldL | *Fjoh_1854* |
| A5FBB0 | 44.64 | 0.59 | Uncharacterized protein | *Fjoh_4505* |
| A5FEA7 | 20.82 | 0.58 | 3-hydroxybutyryl-CoA dehydrogenase | *Fjoh_3448* |
| A5FJS5 | 21.31 | 0.58 | Methylenetetrahydrofolate reductase | *Fjoh_1511* |
| A5FFT4 | 32.63 | 0.57 | Putative thiol-disulphide oxidoreductase DCC | *Fjoh_2915* |
| A5FMT0 | 31.77 | 0.55 | Glycine dehydrogenase (decarboxylating) | *Fjoh_0445* |
| A5FA52 | 20.69 | 0.54 | HPP family protein | *Fjoh_4921* |
| A5FFY4 | 20.77 | 0.54 | Acetolactate synthase small subunit | *Fjoh_2861* |
| A5FIY0 | 22.45 | 0.54 | Phospho-N-acetylmuramoyl-pentapeptide-transferase | *Fjoh_1807* |
| A5FCJ4 | 63.28 | 0.53 | Ribonucleoside-diphosphate reductase, alpha chain | *Fjoh_4065* |
| A5FC99 | 22.55 | 0.53 | Alcohol dehydrogenase GroES domain protein | *Fjoh_4167* |
| A5FNG0 | 57.37 | 0.53 | Glyceraldehyde-3-phosphate dehydrogenase, type I | *Fjoh_0228* |
| A5FFY8 | 20.90 | 0.53 | Pyruvate carboxyltransferase | *Fjoh_2865* |
| A5FIY2 | 28.80 | 0.50 | Cell cycle protein | *Fjoh_1809* |
| A5FNG7 | 52.12 | 0.48 | Candidate beta-glycosyltransferase Glycosyltransferase family 2 | *Fjoh_0221* |
| A5FJ87 | 24.96 | 0.44 | ATP-dependent Clp protease proteolytic subunit | *Fjoh_1702* |
| A5FKI6 | 31.57 | 0.36 | NADH dehydrogenase subunit E | *Fjoh_1242* |
| A5FAQ3 | 40.20 | 0.28 | L-serine dehydratase | *Fjoh_4709* |
| A5FBX4 | 38.52 | 0.12 | Outer membrane efflux protein | *Fjoh_4293* |
