# Supplementary material for: LolA and LolB are conserved in Bacteroidota and are crucial for gliding motility and Type IX secretion
Source: Commun Biol. 2025 Mar 6;8:376. doi: 10.1038/s42003-025-07817-2 (PMC11885536; doi:10.1038/s42003-025-07817-2)
Supplement: Supplementary file 8 — Reporting Summary [file 42003_2025_7817_MOESM8_ESM.pdf]

nature portfolio

Corresponding author(s): Francesco Renzi

Last updated by author(s): Feb 20, 2025

Reporting Summary

Nature Portfolio wishes to improve the reproducibility of the work that we publish. This form provides structure for consistency and transparency in reporting. For further information on Nature Portfolio policies, see our Editorial Policies and the Editorial Policy Checklist.

Statistics

For all statistical analyses, confirm that the following items are present in the figure legend, table legend, main text, or Methods section.

n/a	Confirmed	
☐	☒	The exact sample size (*n*) for each experimental group/condition, given as a discrete number and unit of measurement
☐	☒	A statement on whether measurements were taken from distinct samples or whether the same sample was measured repeatedly
☐	☒	The statistical test(s) used AND whether they are one- or two-sided<br>*Only common tests should be described solely by name; describe more complex techniques in the Methods section.*
☒	☐	A description of all covariates tested
☐	☒	A description of any assumptions or corrections, such as tests of normality and adjustment for multiple comparisons
☐	☒	A full description of the statistical parameters including central tendency (e.g. means) or other basic estimates (e.g. regression coefficient) AND variation (e.g. standard deviation) or associated estimates of uncertainty (e.g. confidence intervals)
☐	☒	For null hypothesis testing, the test statistic (e.g. *F*, *t*, *r*) with confidence intervals, effect sizes, degrees of freedom and *P* value noted<br>*Give P values as exact values whenever suitable.*
☒	☐	For Bayesian analysis, information on the choice of priors and Markov chain Monte Carlo settings
☒	☐	For hierarchical and complex designs, identification of the appropriate level for tests and full reporting of outcomes
☒	☐	Estimates of effect sizes (e.g. Cohen's *d*, Pearson's *r*), indicating how they were calculated

Our web collection on statistics for biologists contains articles on many of the points above.

Software and code

Policy information about availability of computer code

Data collection

- In silico search for LolA and LolB homologs: HHSearch suite 3; in-house Perl script will be available upon request.
- In silico modeling and characterization of LolA and LolB homologs: I-TASSER; AlphaFold 3; PROPKA 3.2 and PDB2PQR 3.6.2 from the AMBER24 force field; Adaptive Poisson-Boltzmann Solver suite 3.4.1
- Amylase activity assays and Verification of the purity of the membrane fractions: SoftMax Pro 7.1
- Mass Spectrometry: Hystar 6.1; timsControl 2.0
- Growth and OM stress tolerance assays: BioTek Gen5 3.05
- Phase-contrast and immunofluorescence microscopy: Zen Pro 3.9
- Transmission Electron Microscopy: Item of Olympus
- Detection of His-tagged LolB variants and SiaC by Western-Blot: GE Healthcare Amersham Imager 600

Data analysis

- In silico modelling and characterization of LolA and LolB homologs: PyMOL Molecular Graphics System Version 1.2r3pre; Color h script of the PyMOL software.
- PEAKS proteomic analysis: PEAKS Studio 11
- In silico characterization of the proteins identified by label-free mass spectrometry: SignalP 6.0; PSORTb 3.0.3; MicroScope platform v3.17.12
- Phase-contrast and immunofluorescence microscopy: Zen Pro 3.9; Microsoft Office PowerPoint
- Detection of His-tagged LolB variants and SiaC by Western-Blot: GE Healthcare Amersham Imager 600; ImageJ 2.14.0
- Data processing using Scaffold for protein identification: Mascot version 2.7.0; Scaffold version 4.11.0; ProteinProphet
- Conservation of LolA and LolB in Bacteroidetes: Delta-blast; phyloT v2.
- Graph generation and statistical analyses: GraphPad Prism version 8.0.1
- Microscopic observations of cell movement on agar pads: Microsoft Clipchamp version 3.1.

For manuscripts utilizing custom algorithms or software that are central to the research but not yet described in published literature, software must be made available to editors and reviewers. We strongly encourage code deposition in a community repository (e.g. GitHub). See the Nature Portfolio guidelines for submitting code & software for further information.

Data

Policy information about availability of data

All manuscripts must include a data availability statement. This statement should provide the following information, where applicable:

- Accession codes, unique identifiers, or web links for publicly available datasets
- A description of any restrictions on data availability
- For clinical datasets or third party data, please ensure that the statement adheres to our policy

Label free proteomics mass spectrometry data are publicly available in the ProteomeXchange Consortium via the PRIDE partner repository with the dataset identifier PXD056078 and 10.6019/PXD056078.
The mass spectrometry proteomics data for E.coli LolA and LolB and F. johnsoniae LolA1 and LolB1 heterologous expression are publicly available in the ProteomeXchange Consortium via the PRIDE partner repository with the dataset identifier PXD059642 and 10.6019/PXD059642.

Research involving human participants, their data, or biological material

Policy information about studies with human participants or human data. See also policy information about sex, gender (identity/presentation), and sexual orientation and race, ethnicity and racism.

Reporting on sex and gender

Use the terms sex (biological attribute) and gender (shaped by social and cultural circumstances) carefully in order to avoid confusing both terms. Indicate if findings apply to only one sex or gender; describe whether sex and gender were considered in study design; whether sex and/or gender was determined based on self-reporting or assigned and methods used.
Provide in the source data disaggregated sex and gender data, where this information has been collected, and if consent has been obtained for sharing of individual-level data; provide overall numbers in this Reporting Summary. Please state if this information has not been collected.
Report sex- and gender-based analyses where performed, justify reasons for lack of sex- and gender-based analysis.

Reporting on race, ethnicity, or other socially relevant groupings

Please specify the socially constructed or socially relevant categorization variable(s) used in your manuscript and explain why they were used. Please note that such variables should not be used as proxies for other socially constructed/relevant variables (for example, race or ethnicity should not be used as a proxy for socioeconomic status).
Provide clear definitions of the relevant terms used, how they were provided (by the participants/respondents, the researchers, or third parties), and the method(s) used to classify people into the different categories (e.g. self-report, census or administrative data, social media data, etc.)
Please provide details about how you controlled for confounding variables in your analyses.

Population characteristics

Describe the covariate-relevant population characteristics of the human research participants (e.g. age, genotypic information, past and current diagnosis and treatment categories). If you filled out the behavioural & social sciences study design questions and have nothing to add here, write "See above."

Recruitment

Describe how participants were recruited. Outline any potential self-selection bias or other biases that may be present and how these are likely to impact results.

Ethics oversight

Identify the organization(s) that approved the study protocol.

Note that full information on the approval of the study protocol must also be provided in the manuscript.

Field-specific reporting

Please select the one below that is the best fit for your research. If you are not sure, read the appropriate sections before making your selection.

☒ Life sciences ☐ Behavioural & social sciences ☐ Ecological, evolutionary & environmental sciences

For a reference copy of the document with all sections, see nature.com/documents/nr-reporting-summary-flat.pdf

Life sciences study design

All studies must disclose on these points even when the disclosure is negative.

Sample size	For each experiment, sample size is referenced in the Material and Methods section
Data exclusions	No data were excluded from the analyses
Replication	Data replication was successful
Randomization	The tested organisms were bacterial cultures, which cannot be individually allocated into experimental groups
Blinding	This is not relevant for this study, since the tested microorganisms could not be individually allocated to experimental groups

Reporting for specific materials, systems and methods

We require information from authors about some types of materials, experimental systems and methods used in many studies. Here, indicate whether each material, system or method listed is relevant to your study. If you are not sure if a list item applies to your research, read the appropriate section before selecting a response.

Materials & experimental systems

n/a	Involved in the study	
☐	☒	Antibodies
☒	☐	Eukaryotic cell lines
☒	☐	Palaeontology and archaeology
☒	☐	Animals and other organisms
☒	☐	Clinical data
☒	☐	Dual use research of concern
☒	☐	Plants

Methods

n/a	Involved in the study	
☒	☐	ChIP-seq
☒	☐	Flow cytometry
☒	☐	MRI-based neuroimaging

Antibodies

Antibodies used	- Rabbit anti-His polyclonal antibodies supplied by MBL, catalog number PM032, lot number 017<br>- Rabbit anti-GroEL polyclonal antibodies supplied by Sigma-Aldrich, catalog number G6532<br>- Custom rabbit anti-SiaC serum supplied by CER Groupe Belgium<br>- Swine-HRP anti-rabbit polyclonal antibodies supplied by Dako, catalog number P0217, lot number 200
Validation	- Rabbit anti-GroEL polyclonal antibodies validated in E. coli

Plants

Seed stocks	*Report on the source of all seed stocks or other plant material used. If applicable, state the seed stock centre and catalogue number. If plant specimens were collected from the field, describe the collection location, date and sampling procedures.*
Novel plant genotypes	*Describe the methods by which all novel plant genotypes were produced. This includes those generated by transgenic approaches, gene editing, chemical/radiation-based mutagenesis and hybridization. For transgenic lines, describe the transformation method, the number of independent lines analyzed and the generation upon which experiments were performed. For gene-edited lines, describe the editor used, the endogenous sequence targeted for editing, the targeting guide RNA sequence (if applicable) and how the editor was applied.*
Authentication	*Describe any authentication procedures for each seed stock used or novel genotype generated. Describe any experiments used to assess the effect of a mutation and, where applicable, how potential secondary effects (e.g. second site T-DNA insertions, mosiacism, off-target gene editing) were examined.*
